# Inhibition of the Inositol Kinase Itpkb Augments Calcium Signaling in Lymphocytes and Reveals a Novel Strategy to Treat Autoimmune Disease

**DOI:** 10.1371/journal.pone.0131071

**Published:** 2015-06-29

**Authors:** Andrew T. Miller, Carol Dahlberg, Mark L. Sandberg, Ben G. Wen, Daniel R. Beisner, John A. H. Hoerter, Albert Parker, Christian Schmedt, Monique Stinson, Jacqueline Avis, Cynthia Cienfuegos, Mark McPate, Pamela Tranter, Martin Gosling, Paul J. Groot-Kormelink, Janet Dawson, Shifeng Pan, Shin-Shay Tian, H. Martin Seidel, Michael P. Cooke

**Affiliations:** 1 The Genomics Institute of the Novartis Research Foundation (GNF), San Diego, California, United States of America; 2 Novartis Pharmaceuticals UK Limited, Respiratory Disease Area, Horsham, West Sussex, United Kingdom; 3 Novartis Institutes for Biomedical Research, Musculoskeletal Disease Area, Basel, Switzerland; 4 Novartis Pharma AG, Novartis Institutes for Biomed. Research, Basel, Switzerland; Jackson Laboratory, UNITED STATES

## Abstract

Emerging approaches to treat immune disorders target positive regulatory kinases downstream of antigen receptors with small molecule inhibitors. Here we provide evidence for an alternative approach in which inhibition of the negative regulatory inositol kinase Itpkb in mature T lymphocytes results in enhanced intracellular calcium levels following antigen receptor activation leading to T cell death. Using Itpkb conditional knockout mice and LMW Itpkb inhibitors these studies reveal that Itpkb through its product IP4 inhibits the Orai1/Stim1 calcium channel on lymphocytes. Pharmacological inhibition or genetic deletion of Itpkb results in elevated intracellular Ca^2+^ and induction of FasL and Bim resulting in T cell apoptosis. Deletion of Itpkb or treatment with Itpkb inhibitors blocks T-cell dependent antibody responses *in vivo* and prevents T cell driven arthritis in rats. These data identify Itpkb as an essential mediator of T cell activation and suggest Itpkb inhibition as a novel approach to treat autoimmune disease.

## Introduction

Lymphocytes become pathogenic when tolerance to self-tissues is lost, often resulting in the onset of autoimmune disease. Receptors that trigger immune cell activation are linked to phosphorylation events mediated by kinases, which has led to the idea that blocking kinases involved in cell activation with low molecular weight inhibitors (LMW) may be an effective way to treat autoimmune disease. Several kinase inhibitors are currently in clinical trials for numerous autoimmune indications, such as rheumatoid arthritis, inflammatory bowel disease, and psoriasis [1]. Current therapies such as Neoral (cyclosporin) and Prograf (FK506) target T lymphocytes by blocking the Ca^2+^-activated phosphatase, calcineurin, which mediates the dephosphorylation of NF-AT, a key transcription factor which drives effector gene expression and other canonical features of lymphocyte function [2]. In mature lymphocytes, the spatio-temporal regulation of Ca^2+^ modulates signaling pathways that serve to control a variety of cellular responses, such as activation, differentiation, effector function, and cell death [3,4]. Thus, targeting kinases involved in Ca^2+^ responses is an attractive approach to treat immune disorders.

The activation of T lymphocytes through the T cell antigen receptor (TCR) results in PLCγ-mediated hydrolysis of phosphatidylinositol 4,5 bisphosphate (PIP_2_) into IP_3_ and diacylglycerol. IP_3_ induces the release of Ca^2+^ from endoplasmic reticulum (ER) stores and the subsequent influx of Ca^2+^ into the cytosol through store-operated Ca^2+^ (SOC) channels in the plasma membrane following store depletion. Two key components of the SOC channel pathway in lymphocytes, often referred to as calcium-release activated calcium (CRAC) channels, were identified as stromal interaction molecule 1 (Stim1) and Orai1. Stim1 senses Ca^2+^ depletion in the ER via its EF hand and subsequently translocates to the plasma membrane where it interacts with Orai1, a pore subunit of the CRAC channels that ultimately permits a sustained increase in cytosolic Ca^2+^ [3–7]. While much of the signaling machinery leading to the induction of Ca^2+^ entry in lymphocytes have been identified, the components and mechanisms by which Ca^2+^ is negatively regulated are incompletely understood.

The soluble second messenger, IP_3_, can be further phosphorylated by inositol kinases into higher order inositol phosphates [8]. The Ca^2+^-dependent kinase, inositol trisphosphate 3’ kinase B (Itpkb), phosphorylates the 3’ position of Ins(1,4,5)P3 to generate inositol 1,3,4,5-tetrakisphosphate [Ins(1,3,4,5)P4] [9]. The importance of Itpkb, and consequently Ins(1,3,4,5)P4, in the immune system is exemplified by Itpkb-deficient mice which lack peripheral T cells due to a block at the CD4^+^CD8^+^ stage of thymocyte development [10,11]. In addition, B cell development is significantly impaired [12,13]. Itpkb-deficient thymocytes and B cells exhibit enhanced SOC channel activity following antigen receptor stimulation, which is reversed upon treatment with exogenous Ins(1,3,4,5)P4. Furthermore, upon thapsigargin treatment, a condition that artificially opens SOC channels independent of other inositol phosphates, Ins(1,3,4,5)P4 is able to inhibit channel activity [13]. These data indicate that Itpkb, and its product Ins(1,3,4,5)P4, are crucial negative regulators of SOC channels in lymphocytes, however, the precise mechanism is unknown [12,13]. Due to the block in thymocyte development and defects in B cell development, the role of Itpkb in mature lymphocytes is also not known.

To elucidate the role of Itpkb in mature lymphocytes, we generated an inducible Itpkb knock-out mouse line using the tamoxifen-induced ERT2-Cre/LoxP system. Here we show that in addition to playing a critical role in lymphocyte development, Itpkb is required for mature T cell survival. *In vivo*, conditional Itpkb-knockout mice fail to generate antibody responses to a T-dependent antigen, while T-independent antibody responses remain intact. Itpkb-deficient T cells fail to proliferate following TCR stimulation; and TCR-mediated SOC responses are enhanced. Electrophysiological analysis revealed that the product of Itpkb, Ins(1,3,4,5)P4 rapidly inhibits Orai1-mediated current in intact cells. TCR stimulation of Itpkb-deficient T cells leads to enhanced expression of genes involved in apoptosis, such as Bim and FasL, and blocking the Fas-FasL interaction partially rescued the observed proliferative defect. To explore the therapeutic potential of targeting Itpkb for T cell-mediated autoimmune diseases, a selective, potent, and orally bioavailable LMW inhibitor of Itpkb, GNF362, was identified via high-throughput compound screening and optimized for drug-like properties using medicinal chemistry. Application of GNF362 to lymphocytes *in vitro*, blocked Ins(1,3,4,5)P4 production, enhanced antigen receptor-driven Ca^2+^ responses and lead to apoptosis of activated T cells in an Itpkb-dependent manner. Mice treated with GNF362 recapitulated the block in T cell development observed in *Itpkb* ^*–/–*^ animals. Lastly, GNF362 significantly inhibited joint swelling and secondary antibody responses in the rat antigen-induced arthritis (rAIA) model. These data identify Itpkb and IP4 as essential mediators of T cell activation and suggest a novel paradigm for the treatment of autoimmune disease in which blockade of the negative regulator Itpkb can drive pathogenic T cells to apoptosis via enhancement of Ca^2+^ signals.

## Materials and Methods

### Mice

All animal work was approved by the Genomics Institute of the Novartis Research Foundation (GNF) Institutional Animal Care and Use Committee (IACUC) committee. All mice were maintained in a specific pathogen-free facility at the GNF. Itpkb-deficient mice (*MsTless*) on a C57BL/6 background were generated as previously described [11]. Conditional Itpkb-deficient mice (*Itpkb* ^*fl/fl*^) were generated by constructing a targeting vector containing exon 2 of Itpkb flanked with two loxP sites (S1 Fig). A neomycin-resistant gene cassette located within the two loxP sites was flanked by FRT sites. Following confirmation of gene targeting in CJ7 ES cells we generated chimeric mice by injecting targeted ES cells into C57BL/6-Tyr(c-2J) blastocysts and implanting the embryos into pseudopregnant CD1 (ICR) mice. Male chimeric mice were bred with the FLPeR female mice to delete the neomycin cassette in vivo. The *Itpkb* floxed mice (*Itpkb* ^*fl/fl*^) were then bred with the ROSA26^Cre−ERT2/+^ transgenic mice, which express Cre recombinase fused to a mutated estrogen receptor ligand-binding domain, to ultimately generate *Itpkb* ^*fl/fl*^ ERT2-Cre transgenic mice whereby Itpkb can be inducibly deleted following treatment with Tamoxifen. Itpkb was inducibly deleted upon treatment with tamoxifen for 5 days, followed by 7 days of rest prior to experimental use. Since ER-Cre is expressed in all tissues, the efficiency of Itpkb could be assessed by PCR of genomic tail DNA. At the genomic level, the efficiency of deletion was estimated to be approximately 90%. These results indicate that Tamoxifen-induced Cre expression induced efficient deletion of Itpkb in all cells. This method of Itpkb deletion was used in all experiments presented here. All of the mice used for experiments presented in this paper were between 6 to 14 weeks of age and between 1 and 4 generations backcrossed to the C57BL/6 background. Mice which were backcrossed at least 4 generations exhibited 95% C57BL/6 genome contribution.

### Genotyping of *Itpkb* ^*fl/fl*^ mice

Mice were genotyped by polymerase chain reaction (PCR) to assess floxed allele, ERT2 Cre transgene, FLPe-ed and Cre deletion post TM treatment. As shown in S2A Fig, six genotyping primers were used to confirm WT and *Itpkb* ^*fl/fl*^ genotype: LAH-F (gcccacattcacaaaacacacttg), Flox-F (aagcccagagtggtaagtgttgc), Neo-F (ttgacgagttcttctgagcgggactctgg), SAH-R (gcccacaacaaaaccacctatagag), CreF315 (tgatgacggtgaacgtgaaa) and CreR647 (ctcgaccagtttagttaccc). PCR amplification conditions were 94°C for 3 min, 33 cycles at 94°C for 20 sec, 60°C for 30 sec and 72°C for 45 sec, then 72°C for 5 min and hold at 10°C. Representative genotyping assays are shown in S2B Fig.

### FACS Analysis

Spleens and thymuses were removed from *Itpkb* ^*+/+*^ and *Itpkb* ^*fl/fl*^ mice, pretreated with tamoxifen for 5 days and allowed to rest for 3–7 days. Following RBC lysis, cells (1-5x10e^5^ cells/well) were washed in cold FACS buffer (1% FBS/2mM EDTA/PBS), blocked with 50 μl Fc Block (BD) for 5–10 min on ice, then surface stained with either CD3-PB (BD), CD8-FITC (BD), B220-PE (BD), IgM-APC (BD) and CD4-APC-Cy7 (BioLegend) for splenocyte staining or CD3-PB (BD), CD69-FITC (BD), TCR-beta (BD), CD4-APC (eBioscience) and CD8-APC-Cy7 (BioLegend) for thymocyte staining. Cells were stained for 30–60 minutes on ice, protected from light. Cells were washed twice and resuspended in either 150 μl FACS buffer to read immediately or BD stabilizer Fixative (BD) for later analysis on the BD LSR II flow cytometer.

### Immunizations

*Itpkb* ^*+/+*^ and *Itpkb* ^*fl/fl*^ mice were immunized intraperitoneal (i.p.) with either 100 μl DNP-KLH/Alum (Calbiochem) at 1mg/ml to assess T cell-dependent antibody responses, or with 100 μl TNP-(27)-AECE-Ficoll (BioSearch Technologies) to assess T cell-independent responses. Blood was obtained from mice before injection and post-immunization on day 9 for DNP-KLH and day 11 for TNP-Ficoll. Serum was analyzed by enzyme-linked immunosorbent assay (ELISA). ELISA plates (NUNC) were coated overnight at 4°C with TNP-BSA (BioSearch Technologies) at 50 μg/ml bi-carbonate buffer. Plates were washed 3 times (0.05% Tween/PBS), blocked with 1% BSA/PBS for 2 h at 25°C, then serial dilutions of serum samples were added, followed by incubation for 2 h at 25°C. Plates were washed six times before adding anti-IgG1-HRP (US Biological, 1/2,000) or anti-IgG3-HRP (Jackson Lab, 1/5000) for 1 h at room temperature. Plates were washed six times, antibodies were detected with Super Aqua Blue substrate (eBioscience), stopped with 0.625 M Oxalic Acid (Fluka) and absorbance was measured at 405 nm.

### Proliferation Assays

Spleens were removed from *Itpkb* ^*+/+*^ and *Itpkb* ^*fl/fl*^ mice. Following RBC lysis, CD4^+^ T cells were purified by negative selection (Miltenyi Biotec) and resuspended in RPMI complete media containing 10% FCS, 2mM L-Glutamine, 100U penicillin, 100 μg/ml streptomycin, 10mM Hepes, 1x NEAA, 1mM Na Pyruvate and 50 μM 2-ME. 5x10^4^ purified CD4^+^ T cells were plated in a 96-well round bottom plate and stimulated with anti-CD3/28 beads (Invitrogen) at either a 0.5:1 or 2.5:1 beads-to-cell ratio. Cells were incubated for 48 h at 37°C, before adding 1μCi/well of ^3^H-thymidine for an additional 18 h at 37°C. 1x10^5^ B cells purified by negative selection (Miltenyi Biotec) were stimulated with anti-IgM (Fab’2), anti-CD40, or LPS at the concentrations indicated in the presence of rIL-4 at 50ng/mL for 72h at 37°C, before adding Cell Titer Glo (Promega). Luminescence was measured on an Acquest fluorescence reader (Molecular Devices).

### Proliferation and Cell Apoptosis assay with CFSE and Annexin V

Purified CD4^+^ T cells from *Itpkb* ^*+/+*^ and *Itpkb* ^*fl/fl*^ mice were labeled with 2.5μM of CFSE (Molecular Probes) in 5%FBS/PBS at 37°C for 10 minutes. Cells were then washed with cold RPMI with 10% FCS, and plated at 1X10^6^cells/ml in 96-well flat bottom plate, stimulated with anti-CD3/28 beads, and incubated at 37°C. To evaluate FasL-mediated cell death, anti-FasL(MFL2) or American Hamster anti-IgG control antibody (eBioscience) at 5 μg/ml final concentration was added to the proliferation assay. After 72h, plates were harvested, washed with cold FACS buffer, and stained with Annexin V-Alexa Fluor647 (BioLegend) followed by 0.1 μg/ml of DAPI (Molecular Probes) and analysis on the flow cytometer.

### Calcium Flux

Methods for analyzing intracellular calcium have been described previously [12,13]. Briefly, splenocytes from *Itpkb* ^*+/+*^ and *Itpkb* ^*fl/fl*^ mice were stained with antibodies to CD4 and B220 prior to be loaded with the calcium sensitive dyes, Fluo-4 and Fura Red. CD4^+^ T cells were stimulated with 10μg/mL of anti-CD3-biotin, followed by 20μg/mL of streptavidin. B220^+^ cells were stimulated 30μg/mL of anti-IgM. For calcium re-addition experiments, 4mM CaCl_2_ was added back. Ionomycin (Sigma) at 1μg was added as a control for dye loading.

### Q-PCR analysis

Purified CD4^+^ T cells from *Itpkb* ^*+/+*^ and *Itpkb* ^*fl/fl*^ mice were stimulated for 0, 2 or 6 hrs with anti-mouse CD3/28 beads (Invitrogen, 2:1 bead to cell ratio). Cells were removed from the plate and total RNA was extracted using the Qiagen QIAshredder and RNeasy Kit. RNA was transcribed into cDNA using the Qiagen QuantiTect Reverse Transcription Kit. Real time qPCR was performed using Applied Biosystem 7900HT Fast Real-Time PCR System, FastStart Universal Probe Master (Rox) and the following Taqman primer probe sets (Applied Biosystems): GAPDH-FAM (Mm99999915_g1), Bcl2-FAM (Mm00477631_m1), Bim or Bcl2l11-FAM (Mm00437796_m1), FasL-FAM (Mm00438864_m1), and Fas-FAM (Mm01204974_m1). To calculate the ∆C_T_ value and to normalize the data to the housekeeping gene (GAPDH), the average threshold cycles (C_T_) values (duplicate wells) for each gene minus the average GAPDH C_T_ value was calculated. The gene expression was then normalized to a no stimulation control (∆C_T_1 - ∆C_T_2 = ∆∆C_T_), followed by fold expression power (2^- ΔΔCT^).

### Patch Clamp

HEK293 cells stably expressing Stim1, and inducible Orai1, were induced with doxycycline for 16–24 hours before experiments on the QPatch HT whole-cell recording system (Sophion, Copenhagen, Denmark). On the experimental day, cells were dissociated and suspended in serum free QPatch medium for up to 4 hours. The intracellular solution was composed of the following (mm): 10 HEPES, 10 BAPTA, 6 MgCl_2_, 120 Glutamic acid, 10 EGTA pH 7.2 with CsOH. The extracellular solutions were composed of the following (mm): 140 NaCl, 10 CaCl_2_, 10 CsCl, 2 MgCl_2_, 2.8 KCl, 10 HEPES, 10 glucose, pH 7.4 with NaOH. Upon cell break-in, the intracellular environment of the cell was dialyzed with the intracellular solution, which chelated free intracellular Ca^2+^ resulting in passive depletion of intracellular Ca^2+^ stores and time-dependent activation of Orai1 current. A voltage ramp from -70 to +70mV was run every 10 seconds with Orai1 currents measured at -70mV from a holding potential of 0mV. The effect of IP4 on Orai1 currents was investigated using either 2,6-Di-O-Butyryl-myo-Inositol 1,3,4,5-Tetrakisphosphate-Octakis (propionoxymethyl) Ester (Bt2-Ins (1,3,4,5)P4/PM), or as a control, Bt2-Ins (1,4,5,6)P4/PM (Sirius Fine Chemicals) at the indicated concentrations. Finally, 30μM of 2-APB was applied at the end of the experiment to block any remaining Orai1 current.

### Kinase Glo Assay

As described previously, Itpkb activity was determined by Kinase-Glo (Promega)[14]. Itpkb inhibitor dose response was carried out in 384well format with 60nM Itpkb, 2μM ATP, 200μM IP3, and 10% FBS at 25C for 1.5hr.

### IP4 HPLC Assay

Jurkat cells were labeled with ^3^H-MyoInositol in inositol free RPMI in the absence of serum for 6–8hrs at 37°C, then resuspended in RPMI with 10% FCS and incubated overnight. Cells were resuspended in the presence of compound prior to the addition of 1μg/mL of OKT3 and 1μg/mL of anti-CD28 for 5 min at 37°C. After stimulation, cells were lysed in PBS with 3% HCL (0.36M final). ^3^H inositols in cellular extracts were resolved by anion exchange HPLC and measured with an in-line β-ram detector.

### Kinase selectivity panel

GNF362 was tested in a panel of 159 protein and lipid kinases using Invitrogen Profiling Z'-lite kinase assay services. Briefly, GNF362 was tested in dose response in the presence of 10 μM ATP. Time-resolved fluorescence resonance energy transfer was utilized as the readout. Data shown represents the percent inhibition observed at 5μM.

### FLIPR Ca^2+^ Assay

Purified wild type splenocytes were labeled with the calcium-sensitive dye, Fluo4, plated onto fibronectin coated 384-well FLIPR plates, and spun at 1000rpm for 4min. Compound was transferred onto FLIPR plate at indicated concentrations, followed by stimulation with 30μg/mL of Fab’2 anti-IgM in the presence of 1mM EGTA. Calcium readings were taken for 7.5min followed by the addition of 5mM Ca^2+^, and SOC-mediated Ca^2+^ entry was measured for the final 5.5 minutes.

### CD4^+^ T cell differentiation and intracellular staining

Purified CD4^+^ T cells from *Itpkb* ^*+/+*^ and *Itpkb* ^*fl/fl*^ mice were stimulated at a 1:1 ratio with anti-CD3/28 beads in Th1- or Th2-skewing conditions. Th1 skewing conditions were anti-IL-4 (eBioscience, 5μg/ml), IL-12 (R&D Systems, 10ng/ml) and IL-2 (Peprotech, at 5ng/ml); Th-2 skewing conditions were IL-4 (R&D Systems, 5ng/ml), anti-IFNγ (eBioscience, 5μg/ml), anti-IL-12 (eBioscience, 4.25μg/ml) and IL-2 (5ng/ml); Cell were cultured at 37°C, for 6 days in the presence of exogenous IL-2 (5ng/ml). On day 6, 100K cells restimulated with anti-CD3/28 beads (~1:1 ratio) for 6 h at 37°C. The final 2h included Golgi stop (BD) and Golgi plug (BD). Cells were then surface stained with anti-CD4- APC-Cy7 (BioLegend), fixed, permeabilized, stained intracellularly with anti-IFNγ-APC, anti-IL4-PE (BioLegend), and anti-IL2-PE-Cy7 (eBioscience), and then run on the flow cytometer.

### In vivo compound studies

GNF362 was formulated at 2mg/mL in 20% hydroxyl propyl-beta cyclodextrin in water (vehicle). Wild type mice were then dosed orally with GNF362 at 3, 10, or 25mg/kg or vehicle twice daily for 9 days. The effect of GNF362 on thymocyte development was assessed by staining thymocytes for CD4-APC and CD8-APC-Cy7, as described above, and determining the percentage of CD4^+^ T cells by FACS analysis.

### Rat AIA model

Lewis rats were immunized on Days − 21 and − 14 by subcutaneous injection of 100 μg methylated bovine serum albumin (mBSA) in 50 μl saline, emulsified in 50 μl complete Freund's adjuvant. GNF362 was then dosed orally at 6 or 20 mg/kg each day until the end of the study. On Day 0, 14d after the second immunization, the rats were given an intra‐articular injection of mBSA (50μg) into the right knee joint. Knee joint swelling was monitored on Days 2, 4, and 7 by measuring knee diameters (mean of four readings), with the joint flexed at an angle of 90°, using a digital micrometer. At the termination of the study, paraffin sections were made and stained with Giemsa and Saffranin O, followed by histological analysis examining inflammatory cell infiltrate, joint damage, and proteoglycan loss scored on a 5-point scaling system. Lastly, serum antibody titers to mBSA were also measured on Day 7 serum.

## Results

### Itpkb is required for T cell development

**Fig 1:**
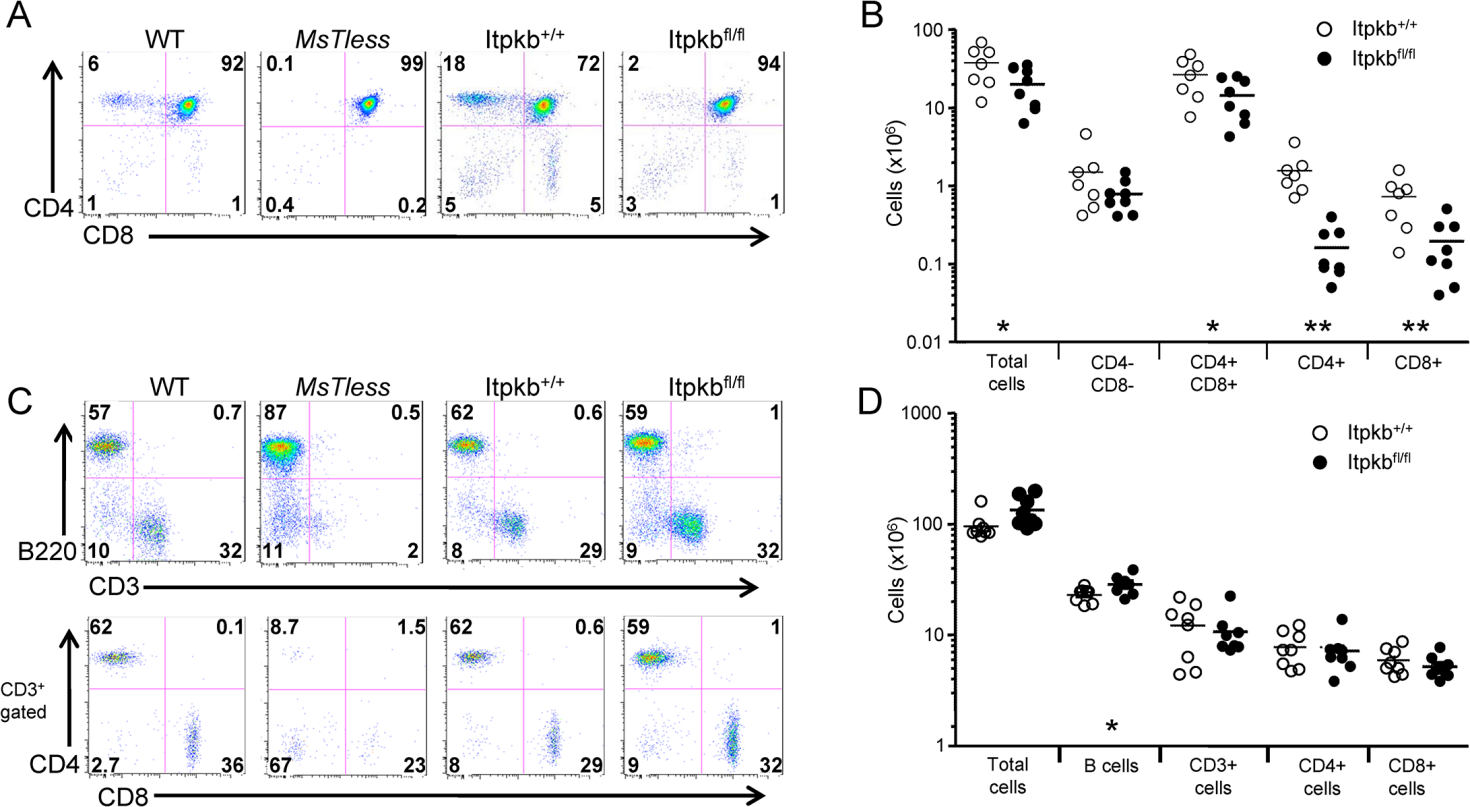
Itpkb is required for T cell development. *Itpkb* ^*+/+*^ and *Itpkb* ^*fl/fl*^ mice were treated with tamoxifen for 5 days followed by 2 days of rest. Thymocytes and splenocytes from tamoxifen-treated mice were compared to WT and *Itpkb* ^*-/-*^ mice via flow cytometry. (A) Gating schemes used for thymocyte analysis with antibodies to CD4, CD8, CD3, and TCRb; numbers in the plots indicate the percentages of each gated population. (B) Total numbers of each thymocyte subset. (C) Gating scheme for analysis of the splenocytes stained with antibodies to CD4, CD8, CD3, and B220; numbers in the plots indicate the percentages of each gated population. (D) Total numbers of the indicated splenocyte populations. DP: double positive. Data from one representative experiment is shown. *, P < 0.05; **, P < 0.01.

To resolve the function of Itpkb in mature lymphocytes, we generated a mouse line whereby Itpkb could be deleted using the tamoxifen-induced ERT2-Cre/LoxP system (S1 and S2 Figs). To determine if inducible deletion of Itpkb disrupts T cell development, the thymic profiles of tamoxifen-treated *Itpkb* ^*fl/fl*^ *Cre* ^*+*^ and control *Itpkb* ^*+/+*^ *Cre* ^*+*^ mice (henceforth referred to as *Itpkb* ^*fl/fl*^ and *Itpkb* ^*+/+*^, respectively) were examined 7 days following tamoxifen treatment and compared with wild type and the ENU-induced mouse mutant, *MsTless*, which possesses an early stop codon in Itpkb [11]. *Itpkb* ^*fl/fl*^ mice contained 10-fold and 5-fold reductions in the percentage of CD4 and CD8 single positive thymocytes while *MsTless* mice display a complete absence of single positive cells in the thymus (Fig 1A). Total numbers of CD4^+^ CD8^+^ double positive thymocytes are significantly reduced in the inducible Itpkb knockout mice (Fig 1B) and these cells fail to upregulate the TCR-associated TCRβ and CD3ε, suggesting impaired TCR signaling and selection (S3A Fig) consistent with previously published data demonstrating a crucial role for Itpkb in thymocyte selection [10,11]. In contrast to the defects in thymocyte number, the percentage and number of B220^+^ and CD3^+^ cells in the spleen of *Itpkb* ^*fl/fl*^ are unchanged (Fig 1C and 1D). These results contrast with dramatic loss of mature T cells observed in *MsTless* mice and suggests that Itpkb is not required for mature lymphocyte survival and homeostasis at this time point. Similar results were observed up to one month following tamoxifen treatment (data not shown).

### Itpkb is required for T cell function by negatively regulating SOC channels

**Fig 2:**
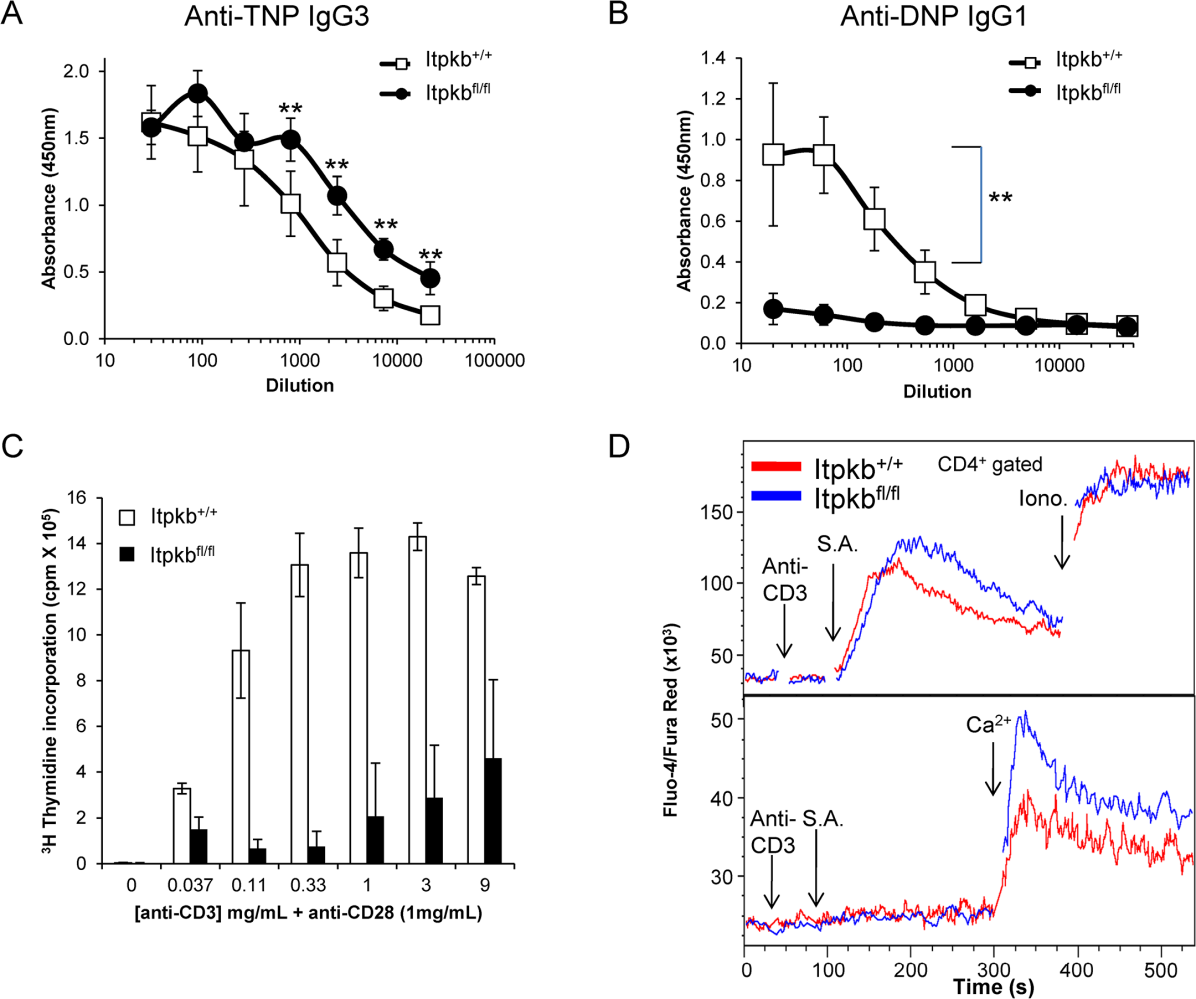
Itpkb is required for T cell function by negatively regulating SOC channels. WT and *Itpkb* ^*fl/fl*^ mice were immunized with either the T-independent antigen, TNP-Ficoll, or the T-dependent antigen DNP-KLH. ELISA of TNP-specific IgG3 (A) or DNP-specific IgG1 (B) antibody responses on day 12 following immunization. Data from one representative experiment is shown (**, P < 0.01). *In vitro*, Itpkb-deficient mature lymphocytes are diminished in their proliferative capacity as measured by thymidine incorporation of purified CD4^*+*^ cells following stimulation with various concentrations of anti-CD3 plus anti-CD28 (C). The data from one representative experiment are shown with values representing the mean counts per minute (cpm) ± SEM. *P < 0.05. Analysis of Ca^*2+*^ responses using the Ca^*2+*^ sensitive dyes Fluo-4 and Fura Red were evaluated after cross-linking the antigen receptor either in the presence or absence of exogenous Ca^*2+*^. Splenocytes gated on CD4 were treated with anti-CD3-biotin, followed by cross-linking with streptavidin (S.A.) in the presence of exogenous calcium (top panel), or in the absence of exogenous calcium, followed by calcium re-addition to examine SOC channel function (bottom panel). Ionomycin (Iono.) stimulation was used at the end of each run to control for equivalent dye loading. Data is shown as the mean fluorescent ratio of Fluo-3 and Fura-Red. Representative data of three independent experiments are shown.

To address the role of Itpkb in mature T lymphocytes, we immunized *Itpkb* ^*fl/fl*^ and control *Itpkb* ^*+/+*^ mice with either a T cell-independent type 2 antigen, TNP-Ficoll, or a T cell-dependent antigen, DNP-KLH. While *Itpkb* ^*fl/fl*^ mice generate slightly elevated IgG3 responses to TNP-Ficoll (Fig 2A), IgG2b responses were normal (S3B Fig), suggesting that Itpkb does not play a major role in the function of mature B lymphocytes to generate an antibody response. In contrast, *Itpkb* ^*fl/fl*^ mice fail to generate IgG1 antibody responses to DNP-KLH (Fig 2B). Furthermore, IgM responses to DNP-KLH were largely unchanged (S4A Fig), demonstrating the dependency on T cells for isotype switching. These data suggest that while Itpkb is not required for mature B cells to mount a T-independent antibody response, it is essential for T cell function in the context of a T-dependent antibody response.

To more closely investigate the role for Itpkb in mature lymphocyte function, purified CD4^+^ T cells from Itpkb-deficient mice were stimulated with anti-CD3/28 and proliferation was assessed. Compared to wild type T cells, Itpkb-deficient CD4^+^ cells possessed a marked reduction in their ability to proliferate, demonstrating a crucial role for Itpkb in the function of mature CD4^+^ T cells (Fig 2C). Reduced proliferative responses were also observed in Itpkb-deficient CD8^+^ cells (data not shown). Interestingly, mature B cells lacking Itpkb respond normally to signals through the B cell receptor (BCR), CD40 and TLR4 (S4B Fig), consistent with the normal T-independent antibody responses.

Prior studies have implicated a role for Itpkb and its product, IP4, in controlling SOC channels in B cells and thymocytes [13]. To determine a role for Itpkb in regulating calcium levels in mature T lymphocytes we examined the calcium response following TCR cross-linking of CD4^+^ T cells from *Itpkb* ^*fl/fl*^ and *Itpkb* ^*+/+*^ mice. Itpkb-deficient CD4^+^ T cells exhibit an increased calcium response compared to wild type cells (Fig 2D, top panel). To determine whether the increased calcium response was due to enhanced SOC entry, CD4^+^ T cells were stimulated with anti-CD3 in the absence of exogenous calcium, followed by the re-addition of calcium. Itpkb-deficient CD4^+^ T cells exhibited increased SOC channel activity (Fig 2D, bottom panel), suggesting that Itpkb and its product, IP4, are required to negatively regulate SOC channels in peripheral CD4^+^ cells. Similarly, Itpkb-deficient B220^+^ cells also demonstrate enhanced SOC channel activity following antigen-receptor stimulation (S4C and S4D Fig). These data indicate that Itpkb is required to negatively regulate SOC signals following the activation of antigen receptors of both mature T and B lymphocytes.

### Itpkb negatively regulates pro-apoptotic gene expression and AICD in CD4^+^ T cells

**Fig 3:**
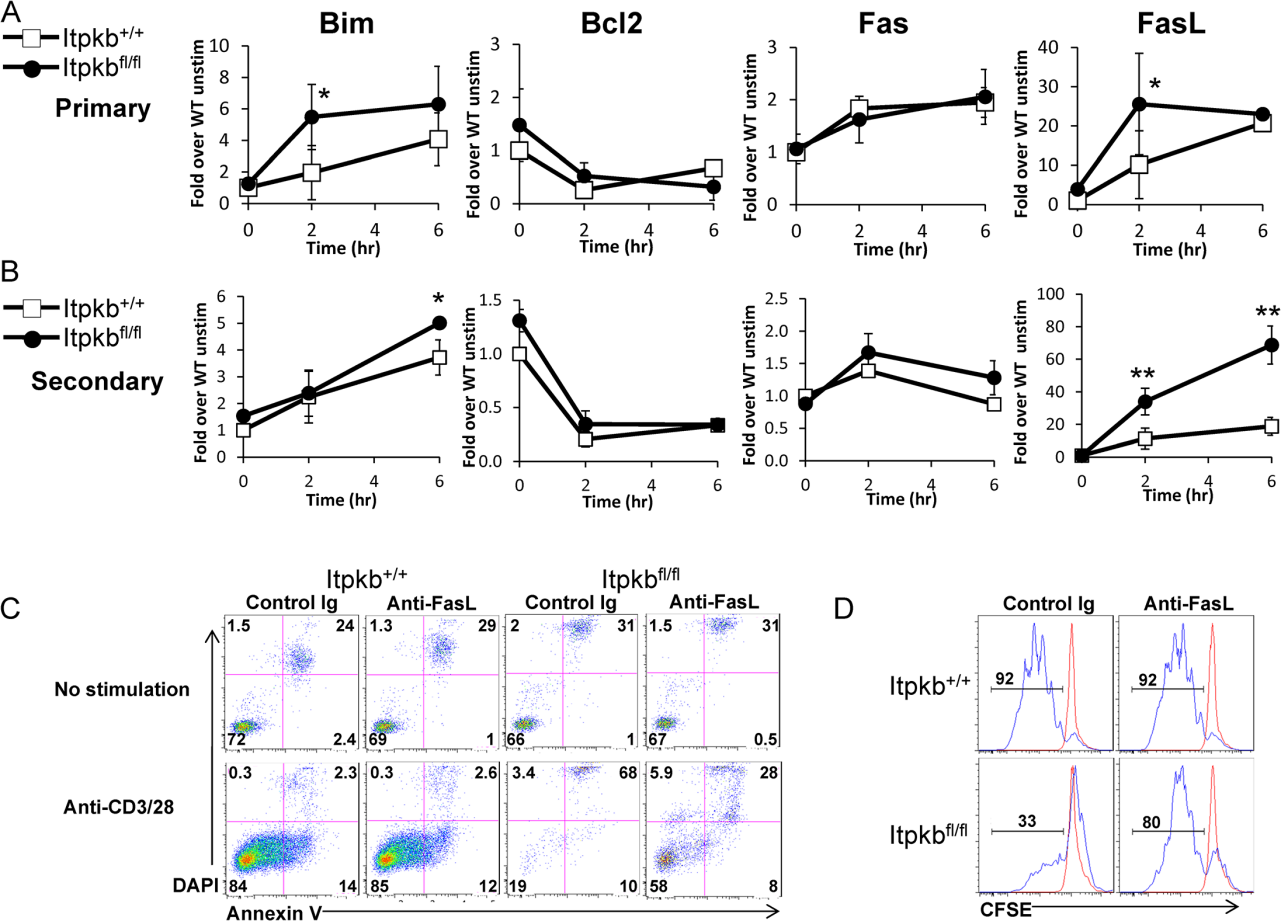
Itpkb negatively regulates pro-apoptotic gene expression and AICD in CD4^*+*^ T cells. Either purified (primary) CD4^*+*^ T cells (A) or in vitro-expanded (secondary) CD4^*+*^ T cells (B) were stimulated with anti-CD3/28 beads for the indicated time points. Following RNA isolation, cDNA was generated, and subjected to real-time quantitative PCR analysis for Bim, Bcl2, Fas, and FasL. The Y-axis represents the respective transcript normalized to either B2-microglobulin or GAPDH values determined for each sample. One representative experiment is shown with the mean values of each genotype ± SE. *, P < 0.05; **, P < 0.01. (C) Purified CD4^*+*^ cells were labeled with CFSE and stimulated with anti-CD3/28 beads in the presence of anti-FasL or an isotype control Ig. 72 hours following stimulation, cells were stained with Annexin V and DAPI. Numbers in the lower left quadrant indicate the percentage of live cells at the time of analysis. (D) Proliferation measured by CFSE dilution was followed after 72 hours in culture. Blue and red lines indicate stimulated and unstimulated cells, respectively. Numbers above bracketed lines indicate the percentage of divided cells. Data shown is representative of three independent experiments.

Prolonged T cell activation results in sustained levels of cytoplasmic Ca^2+^ which drives the expression of pro-apoptotic factors, such as Bim, and death receptor genes, such as FasL, resulting in activation-induced cell death (AICD) to maintain immune homeostasis [15,16] [17]. We reasoned that the enhanced Ca^2+^ signaling in Itpkb-deficient CD4^+^ T cells results in the rapid upregulation of genes involved in AICD. To approach this question, we examined the expression levels of Bim, and the anti-apoptotic factor Bcl2, in comparison to the death receptor-mediated Fas/FasL pathways at 2 and 6 hours following TCR stimulation of primary CD4^+^ T cells. *Itpkb* ^*fl/fl*^ CD4^+^ T cells exhibit enhanced Bim upregulation within 2 hours, whereas Bcl-2 expression levels are largely unaltered compared to control cells. In addition, FasL expression is augmented in *Itpkb* ^*fl/fl*^ CD4^+^ T cells at 2 hours, whereas the expression of Fas is unchanged (Fig 3A). To further confirm these observations, control and *Itpkb* ^*fl/fl*^ CD4^+^ primary T cells were stimulated *in vitro* with PMA and Ionomycin, which bypass the Itpkb defect and allow cells to expand comparably in culture for 5 days, and then re-stimulated through the TCR to determine death effector gene expression levels. While Bim was slightly augmented, FasL expression was augmented 4-fold upon secondary stimulation of T cells from *Itpkb* ^*fl/fl*^ mice (Fig 3B). These data clearly demonstrate the requirement for Itpkb in the negative regulation of gene expression for both death receptor-mediated and mitochondrial-mediated cell death pathways that are critical for proper T cell survival.

To determine if enhanced FasL expression leads to greater AICD in Itpkb-deficient T cells, we stained for Annexin V and DAPI following anti-CD3 plus anti-CD28 stimulation in the presence of a blocking FasL antibody. Prior to stimulation, wild type and Itpkb-deficient T cells exhibit comparable percentages of live (Annexin V^-^ DAPI^-^) cells. Following TCR stimulation, the majority of Itpkb-deficient CD4^+^ T cells die (Annexin V^+^ DAPI^+^) while the viability of wild-type cells is increased. Addition of a blocking anti-FasL antibody resulted in a 3-fold increase in the percentage of viable Itpkb-deficient cells, but did not impact the viability of wild-type cells (Fig 3C). To determine whether blocking FasL is sufficient to restore the proliferative capacity of Itpkb-deficient CD4^+^ T cells, we examined the ability of CFSE-labeled *Itpkb* ^*+/+*^ and *Itpkb* ^*fl/fl*^ CD4^+^ T cells to proliferate to TCR signals in the presence of anti-FasL or a control Ig. The presence of anti-FasL enhanced TCR mediated proliferation of Itpkb-deficient CD4^+^ T cell but did not affect wild type T cell proliferation (Fig 3D). Of note, Itpkb-deficient CD4^+^ T cells rapidly undergo AICD upon TCR stimulation, exemplified by cells becoming Annexin V^+^ before undergoing a single division. This effect is almost completely rescued by the presence of the blocking anti-FasL antibody (S5 Fig). Intracellular cytokine analysis of surviving cells indicated that Itpkb-deficient cells are able to produce similar amounts of IL-2 and Th1 and Th2 cytokines (S6 Fig) following TCR stimulation compared to wild type. Taken together, these data suggest that the primary role of Itpkb is to promote T cell survival via negatively regulating pro-apoptotic factors such as Bim and FasL, which are known to drive AICD.

### The product of Itpkb, Ins(1,3,4,5)P4, inhibits open state Orai1 channels

**Fig 4:**
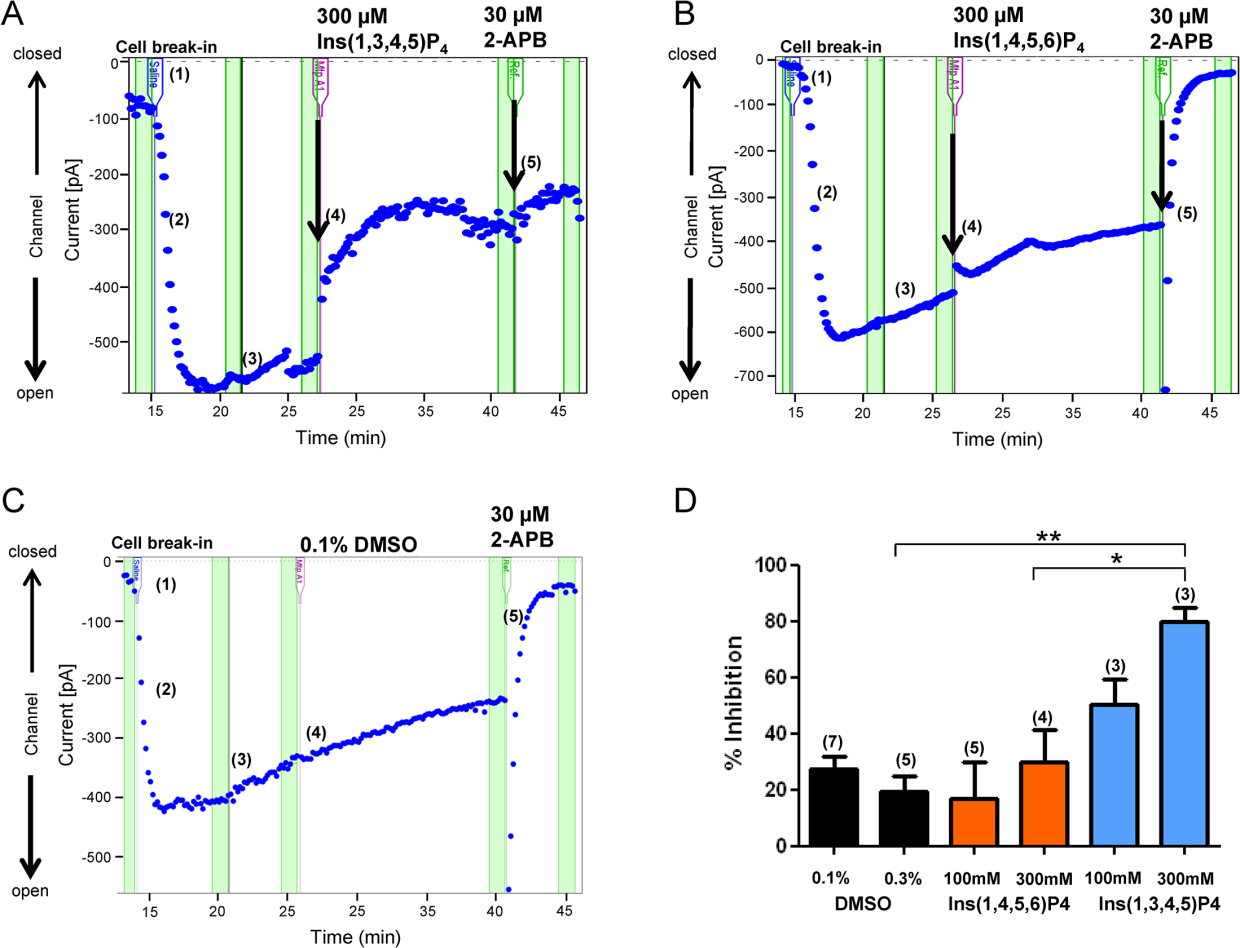
Ins(1,3,4,5)P4 inhibits Orai1-mediated current. HEK293-Orai1-Stim1 cells in serum-free media were placed on the QPatch HT recording system. Following cell break-in (1), little current was measured. Orai1 currents were then activated in a time-dependent manner following passive store depletion with an EGTA/BAPTA solution to chelate intracellular Ca^*2+*^ (2), and then stabilized in an open state (3). Next, (4), Ins(1,3,4,5)P4 (A), a control Ins(1,4,5,6)P4 (B), or 0.1% DMSO (C) was applied for 15 minutes. At the end of each experiment, 2-APB (5) was applied to block any remaining Orai1 current. In (C), percent inhibition values are expressed as the difference in current amplitude before compound application and following 2-APB application (% inhibition = ((base pA–compound pA) / (base pA– 2-APB pA)) X 100. The numbers in parentheses indicate the number of cells tested for each condition on 2 separate experimental days. Data shown is representative of three independent experiments. *, P < 0.05; **, P < 0.01

In order to determine if the product of Itpkb, Ins(1,3,4,5)P4, specifically regulates the main components of the SOC channels in T cells, Orai1 and Stim1, we utilized HEK293 cells which stably express Stim1 and inducibly express Orai1 upon doxycycline treatment. This system has been described to increase SOC entry 20-fold in HEK cells [18]. With the HEK-Stim1-Orai1 cell line, we examined Orai1-mediated currents by whole cell patch clamp. As shown, the chelation of intracellular Ca^2+^ with an EGTA/BAPTA solution stabilizes Orai1 into an open state (Fig 4A–4C; step 2). Following the application of a cell membrane-permeable version of Ins(1,3,4,5)P4, 2,6-di-O-butyryl-myo-inositol 1,3,4,5-tetrakisphosphate-octakis (propionyloxymethyl) ester (Ins(1,3,4,5)P4-PM), 80% of the Orai1 currents are rapidly blocked. The further application of 2-APB, an ion channel blocker, exhibited little effect suggesting almost complete inhibition of the current was achieved by Ins(1,3,4,5)P4 or that Ins(1,3,4,5)P4-PM desensitized 2-APB sensitive channels (Fig 4A and 4D). In contrast, Ins(1,4,5,6)P4, which is not made by Itpkb, as well as the DMSO vehicle did not inhibit Orai1 current (Fig 4B, 4C and 4D). Despite the high concentrations of cell membrane-permeable Ins(1,3,4,5)P4 that were required to observe these effects, these concentrations are comparable to other studies [19–22]. These data demonstrate that the product of Itpkb, Ins(1,3,4,5)P4, is a negative regulator of Orai1-mediated current.

### Identification and characterization of Itpkb inhibitors

**Fig 5:**
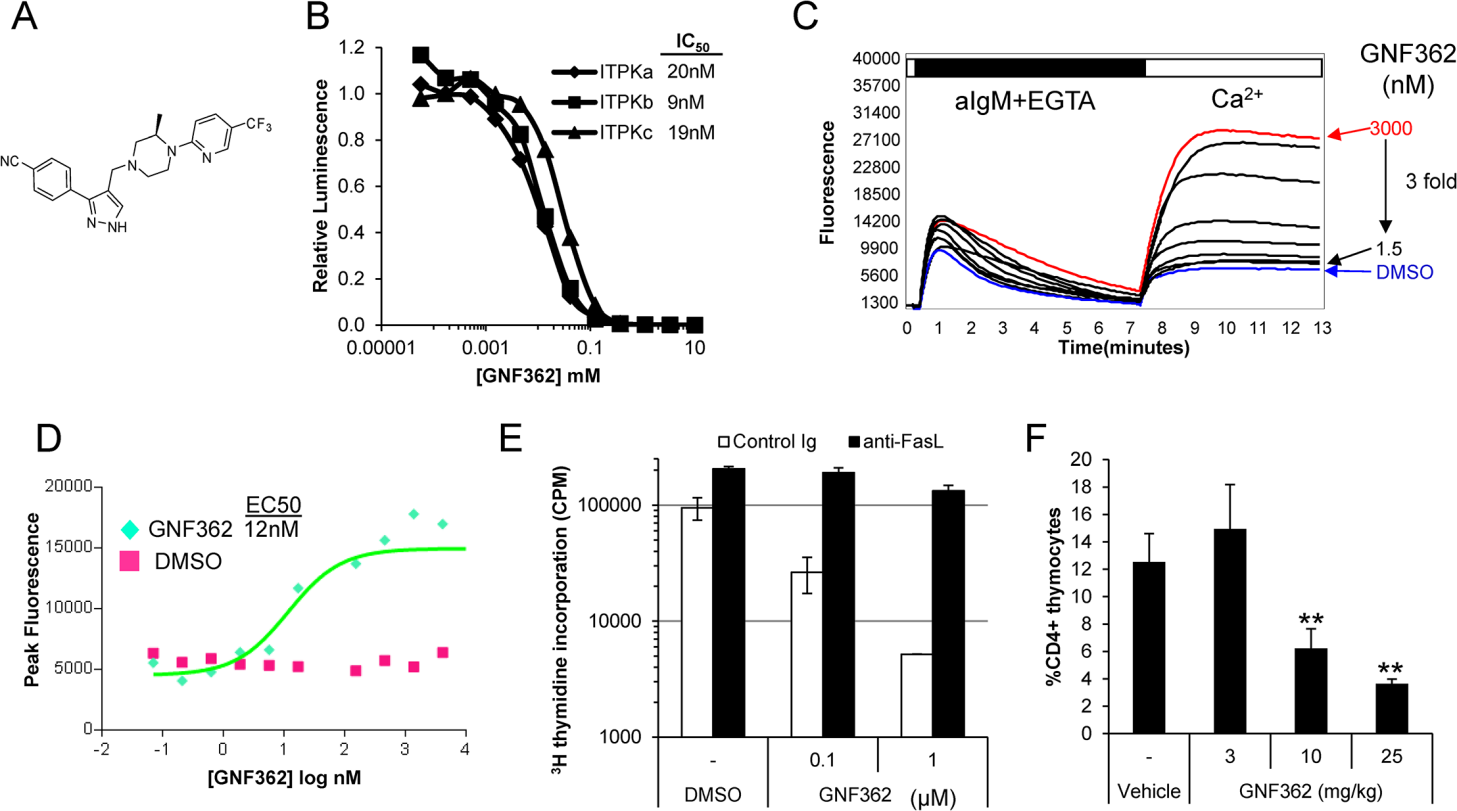
Identification and characterization of Itpkb inhibitors. (A) GNF362 was identified following a 2 million compound biochemical screen, co-crystallography with Itkpb, and additional medicinal chemistry optimization. (B) The biochemical activity of GNF362 was determined in Kinase Glo assays using purified Itpka, Itpkb, and Itpkc proteins [14]. Data shown is one representative experiment. (C) The cellular activity of GNF362 was profiled on wild type B cells loaded with the calcium-sensitive dye, Fluo-4. Splenocytes were pre-incubated with varying concentrations of GNF362, and SOC-mediated Ca^*2+*^ entry, depicted as the mean value of Fluo-4 over time, was measured on the FLIPR following stimulation with anti-IgM and calcium add-back. (D) The peak calcium response, after calcium re-addition is shown as a function of the concentration of GNF362 in the assay, with an EC50 of 12nM. Data shown is one representative experiment. (E) Purified CD4^*+*^ T cells were stimulated in the presence of GNF362, along with the addition of either anti-FasL or a control Ig, to determine the effect on proliferation and FasL-mediated activation-induced cell death. Data shown is representative of three independent experiments. (F) Wild type mice were dosed orally with GNF362 at 3, 10, or 25mg/kg twice daily for 9 days. The percentage of CD4^*+*^ T cells in the thymus was determined by FACS analysis. Data shown is representative of three independent experiments. **, P < 0.01.

The data presented here demonstrate that Itpkb controls mature T lymphocyte function via the regulation of cytosolic Ca^2+^ and the induction of genes involved in apoptosis. We reasoned that blockade of Itpkb with a LMW inhibitor should enhance AICD during the activation and/or effector processes of autoreactive T cells, thus serving as an attractive strategy to eliminate these cells in the treatment of T cell-driven autoimmune disease. To identify a LMW inhibitor of Itpkb, we developed a biochemical assay using purified Itpkb protein to screen a 2 million compound library [14]. Following screening, co-crystallography with Itpkb, and medicinal chemical optimization, we identified GNF362 (Fig 5A), a highly selective and potent orally available LMW inhibitor (IC50 of 9nM), which binds to the ATP-binding pocket of Itpkb (data not shown). GNF362 also potently inhibits Itpka, which is expressed in the brain, as well as Itpkc, which is more broadly expressed (Fig 5B) but has no activity against a panel of more than 150 protein or lipid kinases (S7 Fig). Utilizing a Jurkat cell-based inositol production assay, we found that GNF362 specifically blocked Ins(1,3,4,5)P4 production but had no impact on Ins(1,4,5)P3 production (S8 Fig). When applied to thymocytes, primary B or T lymphocytes, GNF362 augments SOC responses following antigen receptor cross-linking, with an EC50 of 12nM (Fig 5C and 5D; S9A and S9B Fig). The compound has no effect on SOC in Itpkb-deficient cells (S10A Fig) suggesting a high degree of selectivity, and enhances the SOC response of wild-type cells immediately upon addition to activated lymphocytes (S10B Fig).

In order to determine if Itpkb blockade in mature T cells can induce FasL-mediated T cell apoptosis, we examined the ability of T cells to proliferate in the presence of GNF362 as well as a blocking FasL antibody. GNF362 blocked T cell proliferation upon anti-CD3/28 stimulation, and the presence of a blocking anti-FasL antibody reversed this effect, suggesting that GNF362 enhanced FasL-mediated T cell death upon T cell activation (Fig 5E). To understand if blockade of Itpkb disrupts T cell development *in vivo*, mice were dosed orally with GNF362 for 9 days and the percentage of CD4^+^ and CD8^+^ T cells was determined in the thymus and spleen. Treatment with GNF362 lead to a dose dependent reduction in the percentage of CD4^+^ T cells in the thymus (Fig 5F). Similar results were also observed in rats (data not shown). Pharmacokinetic data demonstrate that GNF362 reaches high systemic levels, exhibits moderate volume distribution, with good *in vivo* half-life, and is well tolerated with no adverse events in unmanipulated animals (see additional pharmacokinetic data in S11 Fig). These data indicate that GNF362 selectively inhibits Itpkb, as it recapitulates the characteristics of Itpkb-deficiency.

### Itpkb inhibitors block T cell-driven autoimmune disease

**Fig 6:**
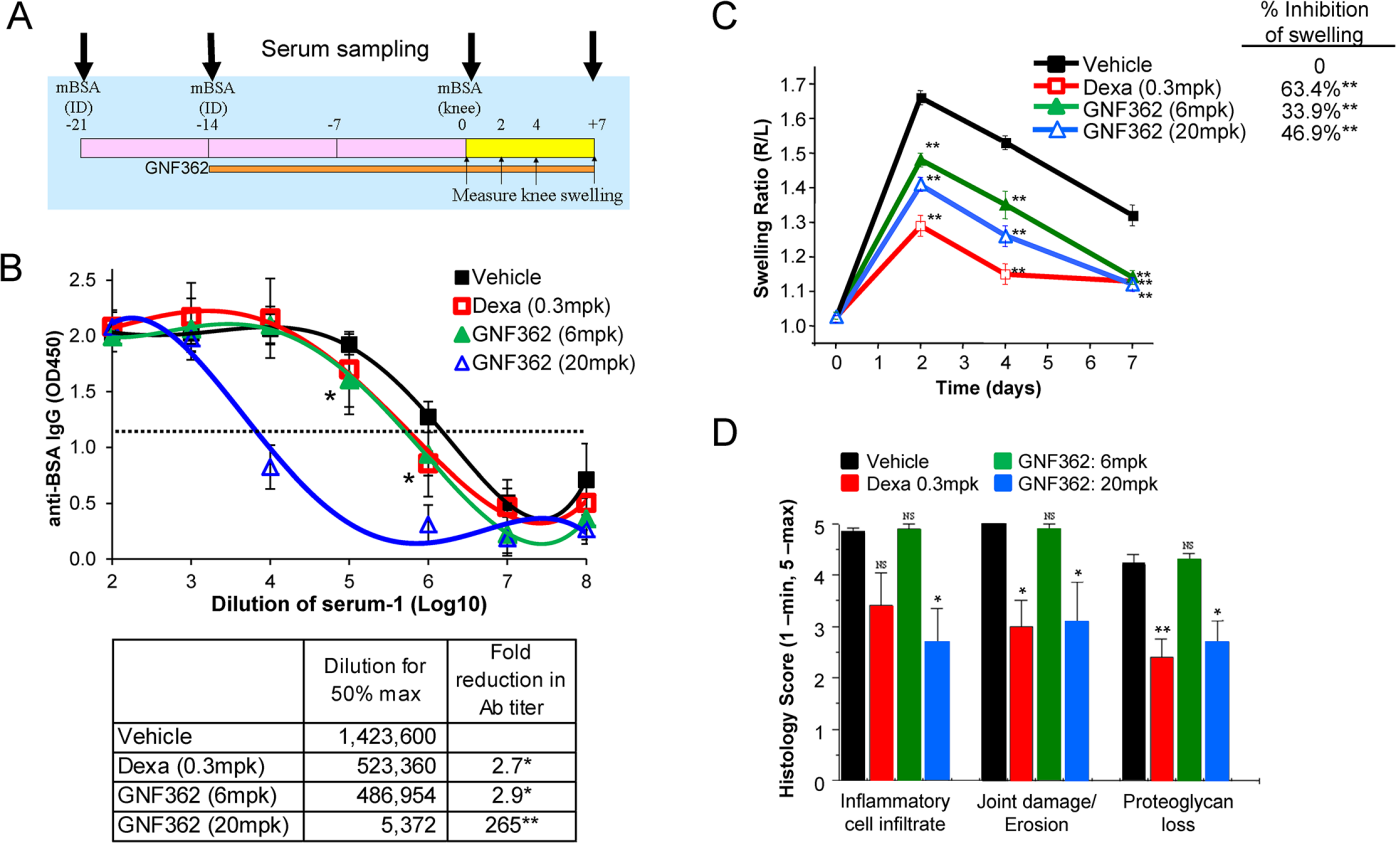
Itpkb inhibitors block rat antigen-induced arthritis. (A) Lewis rats were immunized intra-dermally on Day -21 and -14 with methylated BSA (mBSA), followed by daily oral dosing of GNF362, or dexamethasone (Dex) as a positive control. On Day 0, the rats received an intra-articular injection of mBSA into the right knee joint. (B) Serum was sampled on Day +7, and IgG antibody titers to mBSA were determined by ELISA. The antibody titers were calculated by determining the average dilution at which half-maximal absorbance is detected after subtraction of background. Fold reduction in antibody titer over vehicle is shown in the table. Data shown is one representative experiment. (C) The diameters of the right and left knees were measured on Days 0, 2, 4, and 7, and the ratio of right over left knee diameters (R/L) is shown. (D) Histological analysis of the knee joint was performed blindly and scored on a 5-point scale at the termination of the study. Data shown is one representative experiment. *, P < 0.05; **, P < 0.01.

Rat antigen-induced arthritis (rAIA) is a well-studied animal model of arthritis, due to its similarities with human Rheumatoid Arthritis (RA), and the involvement of T lymphocytes [23]. To determine the efficacy of GNF362 in rAIA, rats were immunized intra-dermally on Day -21 and -14 with methylated BSA (mBSA), followed by daily oral dosing of GNF362, or the steroid-based anti-inflammatory, dexamethasone, as a positive control. On Day 0, the rats received an intra-articular injection of mBSA into the knee joint. Knee joint swelling was measured on Days 2, 4, and 7, followed by histological analysis at the termination of the study. Serum antibody titers to mBSA were also measured on Days -21, -14, 0, and 7 (Fig 6A). Upon repeated immunization with mBSA, antibody levels increase more than 2 million fold compared to unimmunized animals (S12 Fig). Treatment with GNF362 at 20mg/kg beginning on day -14, lead to a 265-fold reduction in antibody titers to mBSA compared to vehicle dosed immunized control animals, indicating that GNF362 can inhibit secondary and tertiary antibody responses (Fig 6B). Furthermore, knee swelling was significantly reduced in both the 20mg/kg and 6mg/kg treatment groups of GNF362 by 47% and 34%, respectively (Fig 6C). Upon histological examination and scoring of the knee joints, GNF362 at 20mg/kg significantly reduced inflammatory cell infiltrate, joint damage, and proteoglycan loss (Fig 6D, S13 Fig). Of note, the 6mg/kg dose of GNF362 shows minimal block in antibody production but shows significant inhibition of joint swelling. These data suggest that blockade of Itpkb by GNF362 could serve as a suitable therapy for rheumatoid arthritis by blocking T cell-mediated inflammation and auto-antibody responses.

## Discussion

The studies described herein test the novel approach of blocking negative regulators of calcium signaling to augment SOC and induce AICD. Utilizing an inducible Itpkb knockout, the current study demonstrates a key role for the inositol kinase, Itpkb, and its product, Ins(1,3,4,5)P4, in blocking mature T lymphocyte death via the inhibition of components of the CRAC machinery, Orai1 and Stim1. To this end, we identified the selective LMW Itpkb inhibitor, GNF362, which not only recapitulated Itpkb-deficiency both *in vitro* and *in vivo*, but blocked disease in a rat model of arthritis. These data suggest that Itpkb may serve as a suitable target for T cell-mediated autoimmune disease.

Previously, we and others have demonstrated that the absence of Itpkb results in a complete block of thymocyte development at the double positive stage [10,11]. In agreement with this finding, we show here that acute deletion of Itpkb in a conditional knockout system, leads to a rapid reduction in the number of single positive thymocytes, further supporting the notion that Itpkb plays a critical role in T cell development. Somewhat surprisingly, inducible deletion of Itpkb does not lead to reductions in peripheral lymphocyte numbers at least over the 1 month timeframe studied. While Itpkb appears dispensable for peripheral lymphocyte homeostasis it is required for T cell function. Immunization of mice following depletion of Itpkb revealed dramatic reductions in T-dependent antibody responses but normal responses to a T-independent antigen. Stimulation of lymphocytes through antigen receptors *in vitro* demonstrate that Itpkb is necessary for the activation of mature T cells through the TCR, but is not required for mature B cells when activated through the BCR. These data indicate that while Itpkb is required for B cell development as reported previously, it may be dispensable for mature B cell function. In contrast, Itpkb is essential for both T cell development and mature T cell function. Interestingly, both mature T and B lymphocytes which have acutely lost Itpkb show enhanced SOC entry following antigen receptor stimulation. Patch-clamp electrophysiology reveals that Ins(1,3,4,5)P4 negatively regulates Orai1 and Stim1, the major components of the SOC machinery in T lymphocytes. Furthermore, TCR stimulation of Itpkb-deficient T cells results in greater induction of both death receptor- and non-death receptor-mediated cell death genes, such as FasL and Bim. Inhibition of FasL function partially restores the ability of Itpkb-deficient T cells to proliferate in response to TCR stimulation implicating FasL as a key mediator of Itpkb-controlled apoptosis.

The current study showing a central role for Itpkb in controlling T cell activation and apoptosis via regulation of Orai1/Stim but not T cell numbers contrast with a recent publication suggesting that Itpkb regulates peripheral T cell number and activation status. In this work, transient overexpression of Itpkb in the thymus of Itpkb^-/-^ mice was used to partially overcome the block in T cell development and lead to the accumulation of low numbers of peripheral T cells that display an activated/memory phenotype[24]. The accumulation of activated memory CD4 cells could reflect homeostatic proliferation, an attempt to control underlying infection or secondary effects of transient high level Itpkb expression in the thymus. It will be interesting to determine the impact of pathogen challenge or long term deletion of inducible *Itpkb* ^*fl/fl*^ animals to resolve these discrepancies.

Numerous reports have described the phenotypes of both Orai1- and Stim1-deficient mice [25–29]. While both *Orai1* ^*-/-*^ and *Stim1* ^*-/-*^ thymocytes exhibit a complete abrogation of SOC entry, the development of αβ^+^ T cells remains intact [25,27,30]. In contrast, while naïve T cells from both Orai1^-/-^ and Stim1^-/-^ mice also display impaired SOC entry, T cell function is significantly impacted as cells fail to induce cytokines upon TCR stimulation [25,27,30]. The comparison of Itpkb-deficient mice to both Orai1- and Stim1-deficient mice reveal an interesting inverse relationship which further validates that these genes act as negative and positive regulators, respectively, within the same signaling pathway. Firstly, while both Orai1-deficient and Stim1-deficient thymocytes and naïve T cells exhibit near complete blocks in SOC entry, Itpkb^-/-^ cells display enhanced SOC entry. In the thymus, Itpkb-deficient mice exhibit a loss of single positive cells, whereas older Orai1-deficient mice (>12–16 weeks) accumulate these cells [26]. In peripheral T cells, Itpkb-deficient T cells exhibit enhanced FasL and Bim expression and FasL-mediated AICD as a result of elevated SOC entry, while Orai1-deficient T cells are resistant to AICD due to a lack of calcium-mediated induction of FasL and other mitochondrial-mediated death genes [26]. Together, these results are consistent with the hypothesis that the fate of developing and mature T cells is controlled by the magnitude of the TCR induced intracellular calcium influx through Orai1 and Stim1 and inhibited by Itpkb-induced production of Ins(1,3,4,5)P4. Since T cell development occurs over a period of days, and T cell activation over a period of hours, these data suggest that the activation threshold required for positive selection versus T cell activation differ greatly and highlight the complexity of the requirement for calcium during various T cell development and activation processes.

One question arising from the study presented here is how Ins(1,3,4,5)P4 regulates the inhibition of Orai1 channels. Ins(1,3,4,5)P4 is thought to function as a soluble analog of phosphatidylinositol 3,4,5-trisphosphate (PIP3) by binding to and potentially competing for the pleckstrin homology (PH) domain of several signaling proteins [31]. Interestingly, it is believed that the redistribution of Stim1 is regulated by the binding of its C-terminal polybasic motif to the negatively charged phospholipids PIP2 and PIP3 in the plasma membrane [3,32–34]. Furthermore, when the polybasic domain of Stim1 is removed in Orai1-expressing cells, enhanced activation of SOC channels is observed [35], suggesting that this domain is required for the negative regulation of SOC channels. Numerous reports have also demonstrated how inhibitors of phosphoinositide synthesis, such as wortmannin, block SOC channel activity that is mediated by Stim1 [34,36].

Several members of the Tec family kinases are recruited to the plasma membrane by binding PIP3 via their PH domain, where they function to activate PLCγ-1, thus permitting the hydrolysis of PIP2 into diacylglycerol and Ins(1,4,5)P3, the substrate of Itpkb [37]. The Tec family members Itk, Btk, and Tec, have been shown to bind Ins(1,3,4,5)P4 via their PH domains [38,39]. While the functional consequence of this interaction remains controversial, one could speculate that Ins(1,3,4,5)P4 may function in a negative feedback loop to inhibit TCR proximal signaling events by promoting the dissociation of PH domain-containing proteins, such as Itk, from the plasma membrane. It is interesting to note that Itk-deficient mice exhibit reduced TCR-mediated calcium entry, resulting in impaired FasL-mediated AICD [40]. With respect to Ins(1,3,4,5)P4-mediated calcium inhibition, it is compelling to speculate that increased levels of Ins(1,3,4,5)P4 may serve to bind to the C-terminal polybasic motif of Stim1 normally required for membrane association and ultimately dissociate the interaction between Orai1 and Stim1. Current efforts are focused on resolving this mechanism.

Blockade of T lymphocyte activation has been a dominant approach to developing novel treatments for autoimmune and inflammatory disorders. While drugs such as Neoral (cyclosporin) and Prograf (FK506) are efficacious they are not free of serious adverse events that limit their use and chronic immune suppression which renders patients susceptible to opportunistic infections [2]. A more desired outcome would be the selective blockade or depletion of pathogenic T lymphocytes while preserving normal T cell function. While several approaches are possible, the data presented here provide proof of concept for a new modality in which auto-reactive T cells are induced to undergo AICD via the elevation of cytoplasmic calcium. Importantly, since AICD requires TCR activation, such compounds may not impact peripheral lymphocyte homeostasis and may not require life-long treatment. Future studies testing the impact of Itpkb inhibition along with specific antigen stimulation may hold promise to achieve selective ablation of pathogenic T cells and provide new therapies to treat autoimmune disease.

## Supporting Information

S1 Fig Generating conditional Itpkb knockout mice. The gene targeting strategy for generation of the Itpkb conditional knockout where mice whose Itpkb exon 2 is flanked by two loxP sequences where crossed to mice transgenic for a tamoxifen-inducible Cre recombinase, to produce *Itpkb* ^*fl/fl*^ *Cre* ^*+*^ mice and *Itpkb* ^*+/+*^ *Cre* ^*+*^ control mice (A). A targeting vector containing LoxP (LP, indicated by triangles) sites flanking Itpkb exon 2 was generated and the structure is shown with the indicated restriction enzyme sites (EcoRI, EI; EcoRV, EV; NotI, NI), Neo cassette, and FRT sites (indicated by ovals). The lengths of the predicted fragments following restriction enzyme digestion are also noted. The complete floxed allele was obtained after excision of the FRT-flanked neo cassette through crossing to FLPeR transgenic mice. The structure of the deleted allele obtained after Cre-mediated excision of the floxed region is also sketched. (B) DNA from the targeted CJ7 ES cells was digested with the indicated restriction enzymes and subjected to southern blot analysis with either a probe recognizing the short arm (SA) or long arm (LA). The short arm of the targeted allele yields a 5kb fragment upon EcoRI digestion. Similarly, the long arm of the targeted allele yields a 17kb fragment upon EcoRV and NotI digestion.
(TIFF)

S2 Fig PCR Genotyping strategy for *Itpkb* ^*fl/fl*^ mice. A schematic of lengths of the expected PCR products (A) are shown. Tail DNA from mice with the indicated genotypes that were either untreated or tamoxifen-treated, and representative PCR results are shown (B).
(TIFF)

S3 Fig Itpkb is required for the upregulation of activation markers on double-positive thymocytes and not required for T-independent antibody responses. (A) Flow cytometry of thymocytes from WT, *Itpkb* ^*-/-*^, *Itpkb* ^*+/+*^ *Cre* ^*+*^, and *Itpkb* ^*fl/fl*^ *Cre* ^*+*^ mice stained with antibodies to CD4, CD8, TCRb, and CD3. CD4^+^CD8^+^ cells were gated, and the percentage of cells expressing TCRb (top) or CD3 (bottom) is shown. The numbers in the plots indicate the percentages of each gated population. (B) Sera from WT and *Itpkb* ^*fl/fl*^ mice that were immunized with the T-independent antigen, TNP-Ficoll in Fig 2A, were tested for TNP-specific IgG2b antibody levels by ELISA on day 12 post-immunization. Data shown are one representative experiment (**, P < 0.01).
(TIFF)

S4 Fig Itpkb-deficient mature B lymphocytes proliferate normally, yet exhibit enhanced SOC entry. (A) B220^+^ cells were stimulated with various concentrations of F(ab’)2 anti-IgM, anti-CD40, or LPS, and proliferation was measured by Cell Titer Glo. (B) Splenocytes gated on B220 were stimulated with F(ab’)2 anti-IgM in the presence of exogenous calcium(C), or in the absence of exogenous calcium, followed by calcium re-addition (D). Data is shown as the mean fluorescent ratio of Fluo-3 and Fura-Red. The data are representative of five independent experiments.
(TIFF)

S5 Fig Itpkb negatively regulates activation-induced cell death of T lymphocytes via FasL. (A) Purified CD4^+^ cells were labeled with CFSE and stimulated with anti-CD3/28 beads in the presence of anti-FasL or an isotype control Ig. 72 hours following stimulation, CFSE dilution versus Annexin V staining was followed to determine whether Annexin V positivity required cell division. Numbers in the top right quadrant indicate the percentage of cells that died prior to cell division. Data shown are representative of four independent experiments.
(TIFF)

S6 Fig Itpkb does not control cytokine production. Itpkb-deficient T cells which survive primary stimulation do not possess any cytokine defects upon secondary stimulation. WT and Itpkb-deficient CD4^+^ T cells were stimulated with anti-CD3/28 beads in either Th1- or Th2-skewing conditions in the presence of exogenous IL-2. After 6 days in culture, live cells were re-stimulated and stained intracellularly for either IL-2 and IFNγ (Th1 cells) or IL-4 (Th2 cells). The bar graph represents the percentage of CD4^+^ cells which are positive for the respective cytokine. Data shown is representative of three independent experiments.
(TIFF)

S7 Fig GNF362 does not exhibit activity on other protein or lipid kinases The activity of GNF362 was tested across a panel of 159 protein and lipid kinases. The percent of kinase inhibition at a concentration of 5μM is shown.
(TIFF)

S8 Fig GNF362 specifically blocks IP4 production. Jurkat T cells were labeled with ^3^H-myo-inositol and activated through the T cell receptor for 5 minutes. The inositol phosphates IP3, IP4, and IP5 were resolved by HPLC using an in-line β-ram detector. Raw HPLC traces from cells stimulated with anti-CD3 + anti-CD28 in the absence or presence of GNF362 are shown in (A). The area under the peaks corresponding to IP4 and IP5 were quantified, and data was normalized to IP5 levels, as this remained unchanged with stimulation. Normalized IP4 levels as a function of GNF362 concentration with an IC50 of 20nM is shown in (B). Data shown is one representative experiment.
(TIFF)

S9 Fig GNF362 enhances SOC entry in thymocytes and mature T lymphocytes. The effect of GNF362 on Ca^2+^ responses was measured using the Ca^2+^ sensitive dyes Fluo-4 and Fura Red following TCR-mediated cross-linking either in the presence or absence of exogenous Ca^2+^. (A) CD4^+^8^+^, CD4^+^, or CD8^+^ thymocytes pre-incubated with DMSO or 1μM of GNF362, were treated with anti-CD3-biotin, followed by cross-linking with streptavidin in the presence of exogenous calcium (left column), or in the absence of exogenous calcium, followed by calcium re-addition to examine SOC channel function (right column). (B) Similarly, CD4^+^ or CD8^+^ splenocytes were stimulated in the same fashion after pre-incubation with GNF362. Data is shown as the mean fluorescent ratio of Fluo-3 and Fura-Red. The data shown are representative of three independent experiments.
(TIFF)

S10 Fig GNF362 rapidly enhances SOC entry in an Itpkb-dependent manner. (A) To demonstrate Itpkb-dependency of GNF362, wild type (top panel) and *Itpkb* ^*-/-*^ (bottom panel) B cells pre-incubated with DMSO or 1μM of GNF362 were stimulated with anti-IgM in the absence of exogenous calcium, followed by calcium re-addition. (B) To examine the effect of GNF362 on SOC entry more directly, wild type B cells were stimulated with IgM in the absence of calcium, followed by calcium re-addition. DMSO or 1μM GNF362 were added to cells after calcium re-addition. Data is shown as the mean fluorescent ratio of Fluo-3 and Fura-Red. The data shown are representative of 3 independent experiments.
(TIFF)

S11 Fig GNF362 exposure following oral dosing. (A) Mice were dosed at 5mg/kg I.V., or 20mg/kg P.O., and bled at various time points. GNF362 levels in plasma were determined by mass spectrometry. Compound clearance (CL), volume distribution (Vss), total exposure (area under the curve, AUC), Cmax, and in vivo half-life (T_1/2_) were calculated. (B) Mice were dosed with GNF362 twice a day (QD) for 20 days at either 6 or 20 mg/kg. Compound levels in plasma were determined at 2, 6, and 24hrs following the last dose. In addition, at the termination of the study, compound levels in the spleen were determined at 24hrs following the last dose. Data is representative of at least five independent experiments.
(TIFF)

S12 Fig Rat AIA model. Repeated immunization with mBSA increases antibody levels. Rats were immunized intra-dermally with mBSA on Days -21 and -14, followed by an intra-articular challenge on Day 0. Antibody titers to mBSA at Days -21, -14, 0, and +7 were determined by ELISA. Data shown is one representative experiment.
(TIFF)

S13 Fig Rat AIA joint histology. Following the termination of the arthritis study, knee joints from each group were removed and paraffin sections were subjected to histological analysis following Giemsa and Saffranin O staining. Representative histopathology data are shown at 1.3x and 5x magnification. Data shown is one representative experiment.
(TIFF)

## References

[1] Kontzias A, Laurence A, Gadina M, O'Shea JJ (2012) Kinase inhibitors in the treatment of immune-mediated disease. F1000 Med Rep 4: 5 10.3410/M4-5 22403586 PMC3297200

[2] O'Neill LA (2006) Targeting signal transduction as a strategy to treat inflammatory diseases. Nat Rev Drug Discov 5: 549–563. 16773072 10.1038/nrd2070

[3] Hogan PG, Lewis RS, Rao A (2010) Molecular basis of calcium signaling in lymphocytes: STIM and ORAI. Annu Rev Immunol 28: 491–533. 10.1146/annurev.immunol.021908.132550 20307213 PMC2861828

[4] Oh-hora M (2009) Calcium signaling in the development and function of T-lineage cells. Immunol Rev 231: 210–224. 10.1111/j.1600-065X.2009.00819.x 19754899

[5] Feske S, Gwack Y, Prakriya M, Srikanth S, Puppel SH, et al (2006) A mutation in Orai1 causes immune deficiency by abrogating CRAC channel function. Nature 441: 179–185. 16582901 10.1038/nature04702

[6] Prakriya M, Feske S, Gwack Y, Srikanth S, Rao A, et al (2006) Orai1 is an essential pore subunit of the CRAC channel. Nature 443: 230–233. 16921383 10.1038/nature05122

[7] Vig M, Kinet JP (2009) Calcium signaling in immune cells. Nat Immunol 10: 21–27. 10.1038/ni.f.220 19088738 PMC2877033

[8] Miller AT, Chamberlain PP, Cooke MP (2008) Beyond IP3: roles for higher order inositol phosphates in immune cell signaling. Cell Cycle 7: 463–467. 18235237 10.4161/cc.7.4.5518

[9] Communi D, Dewaste V, Erneux C (1999) Calcium-calmodulin-dependent protein kinase II and protein kinase C-mediated phosphorylation and activation of D-myo-inositol 1,4, 5-trisphosphate 3-kinase B in astrocytes. J Biol Chem 274: 14734–14742. 10329669 10.1074/jbc.274.21.14734

[10] Pouillon V, Hascakova-Bartova R, Pajak B, Adam E, Bex F, et al (2003) Inositol 1,3,4,5-tetrakisphosphate is essential for T lymphocyte development. Nat Immunol 4: 1136–1143. 14517551 10.1038/ni980

[11] Wen BG, Pletcher MT, Warashina M, Choe SH, Ziaee N, et al (2004) Inositol (1,4,5) trisphosphate 3 kinase B controls positive selection of T cells and modulates Erk activity. Proc Natl Acad Sci U S A 101: 5604–5609. 15064401 10.1073/pnas.0306907101 PMC397439

[12] Miller AT, Beisner DR, Liu D, Cooke MP (2009) Inositol 1,4,5-trisphosphate 3-kinase B is a negative regulator of BCR signaling that controls B cell selection and tolerance induction. J Immunol 182: 4696–4704. 10.4049/jimmunol.0802850 19342645

[13] Miller AT, Sandberg M, Huang YH, Young M, Sutton S, et al (2007) Production of Ins(1,3,4,5)P4 mediated by the kinase Itpkb inhibits store-operated calcium channels and regulates B cell selection and activation. Nat Immunol 8: 514–521. 17417640 10.1038/ni1458

[14] Chamberlain PP, Sandberg ML, Sauer K, Cooke MP, Lesley SA, et al (2005) Structural insights into enzyme regulation for inositol 1,4,5-trisphosphate 3-kinase B. Biochemistry 44: 14486–14493. 16262249 10.1021/bi051256q

[15] Cante-Barrett K, Gallo EM, Winslow MM, Crabtree GR (2006) Thymocyte negative selection is mediated by protein kinase C- and Ca2+-dependent transcriptional induction of bim [corrected]. J Immunol 176: 2299–2306. 16455986 10.4049/jimmunol.176.4.2299

[16] Sandalova E, Wei CH, Masucci MG, Levitsky V (2004) Regulation of expression of Bcl-2 protein family member Bim by T cell receptor triggering. Proc Natl Acad Sci U S A 101: 3011–3016. 14970329 10.1073/pnas.0400005101 PMC365736

[17] Krammer PH, Arnold R, Lavrik IN (2007) Life and death in peripheral T cells. Nat Rev Immunol 7: 532–542. 17589543 10.1038/nri2115

[18] Mercer JC, Dehaven WI, Smyth JT, Wedel B, Boyles RR, et al (2006) Large store-operated calcium selective currents due to co-expression of Orai1 or Orai2 with the intracellular calcium sensor, Stim1. J Biol Chem 281: 24979–24990. 16807233 10.1074/jbc.M604589200 PMC1633822

[19] Bird GS, Putney JW Jr. (1996) Effect of inositol 1,3,4,5-tetrakisphosphate on inositol trisphosphate-activated Ca2+ signaling in mouse lacrimal acinar cells. J Biol Chem 271: 6766–6770. 8636098 10.1074/jbc.271.12.6766

[20] Hermosura MC, Takeuchi H, Fleig A, Riley AM, Potter BV, et al (2000) InsP4 facilitates store-operated calcium influx by inhibition of InsP3 5-phosphatase. Nature 408: 735–740. 11130077 10.1038/35047115

[21] Marechal Y, Pesesse X, Jia Y, Pouillon V, Perez-Morga D, et al (2007) Inositol 1,3,4,5-tetrakisphosphate controls proapoptotic Bim gene expression and survival in B cells. Proc Natl Acad Sci U S A 104: 13978–13983. 17709751 10.1073/pnas.0704312104 PMC1955816

[22] Wilcox RA, Challiss RA, Liu C, Potter BV, Nahorski SR (1993) Inositol-1,3,4,5-tetrakisphosphate induces calcium mobilization via the inositol-1,4,5-trisphosphate receptor in SH-SY5Y neuroblastoma cells. Mol Pharmacol 44: 810–817. 8232232

[23] Holmdahl R, Lorentzen JC, Lu S, Olofsson P, Wester L, et al (2001) Arthritis induced in rats with nonimmunogenic adjuvants as models for rheumatoid arthritis. Immunol Rev 184: 184–202. 12086312 10.1034/j.1600-065x.2001.1840117.x

[24] Pouillon V, Marechal Y, Frippiat C, Erneux C, Schurmans S (2012) Inositol 1,4,5-trisphosphate 3-kinase B (Itpkb) controls survival, proliferation and cytokine production in mouse peripheral T cells. Adv Biol Regul. 10.1016/j.jbior.2012.08.001 22981169

[25] Gwack Y, Srikanth S, Oh-Hora M, Hogan PG, Lamperti ED, et al (2008) Hair loss and defective T- and B-cell function in mice lacking ORAI1. Mol Cell Biol 28: 5209–5222. 10.1128/MCB.00360-08 18591248 PMC2519726

[26] Kim KD, Srikanth S, Yee MK, Mock DC, Lawson GW, et al (2011) ORAI1 deficiency impairs activated T cell death and enhances T cell survival. J Immunol 187: 3620–3630. 10.4049/jimmunol.1100847 21873530 PMC3178683

[27] Oh-Hora M, Yamashita M, Hogan PG, Sharma S, Lamperti E, et al (2008) Dual functions for the endoplasmic reticulum calcium sensors STIM1 and STIM2 in T cell activation and tolerance. Nat Immunol 9: 432–443. 10.1038/ni1574 18327260 PMC2737533

[28] Schuhmann MK, Stegner D, Berna-Erro A, Bittner S, Braun A, et al (2010) Stromal interaction molecules 1 and 2 are key regulators of autoreactive T cell activation in murine autoimmune central nervous system inflammation. J Immunol 184: 1536–1542. 10.4049/jimmunol.0902161 20028655

[29] Vig M, DeHaven WI, Bird GS, Billingsley JM, Wang H, et al (2008) Defective mast cell effector functions in mice lacking the CRACM1 pore subunit of store-operated calcium release-activated calcium channels. Nat Immunol 9: 89–96. 18059270 10.1038/ni1550 PMC2377025

[30] Beyersdorf N, Braun A, Vogtle T, Varga-Szabo D, Galdos RR, et al (2009) STIM1-independent T cell development and effector function in vivo. J Immunol 182: 3390–3397. 10.4049/jimmunol.0802888 19265116

[31] Sauer K, Cooke MP (2010) Regulation of immune cell development through soluble inositol-1,3,4,5-tetrakisphosphate. Nat Rev Immunol 10: 257–271. 10.1038/nri2745 20336153 PMC2922113

[32] Liou J, Fivaz M, Inoue T, Meyer T (2007) Live-cell imaging reveals sequential oligomerization and local plasma membrane targeting of stromal interaction molecule 1 after Ca2+ store depletion. Proc Natl Acad Sci U S A 104: 9301–9306. 17517596 10.1073/pnas.0702866104 PMC1890489

[33] Chvanov M, Walsh CM, Haynes LP, Voronina SG, Lur G, et al (2008) ATP depletion induces translocation of STIM1 to puncta and formation of STIM1-ORAI1 clusters: translocation and re-translocation of STIM1 does not require ATP. Pflugers Arch 457: 505–517. 10.1007/s00424-008-0529-y 18542992 PMC2770109

[34] Walsh CM, Chvanov M, Haynes LP, Petersen OH, Tepikin AV, et al (2010) Role of phosphoinositides in STIM1 dynamics and store-operated calcium entry. Biochem J 425: 159–168. 10.1042/BJ20090884 PMC2860680 19843011

[35] Yuan JP, Zeng W, Dorwart MR, Choi YJ, Worley PF, et al (2009) SOAR and the polybasic STIM1 domains gate and regulate Orai channels. Nat Cell Biol 11: 337–343. 10.1038/ncb1842 19182790 PMC2663385

[36] Broad LM, Braun FJ, Lievremont JP, Bird GS, Kurosaki T, et al (2001) Role of the phospholipase C-inositol 1,4,5-trisphosphate pathway in calcium release-activated calcium current and capacitative calcium entry. J Biol Chem 276: 15945–15952. 11278938 10.1074/jbc.M011571200

[37] Miller AT, Berg LJ (2002) New insights into the regulation and functions of Tec family tyrosine kinases in the immune system. Curr Opin Immunol 14: 331–340. 11973131 10.1016/s0952-7915(02)00345-x

[38] Huang YH, Grasis JA, Miller AT, Xu R, Soonthornvacharin S, et al (2007) Positive regulation of Itk PH domain function by soluble IP4. Science 316: 886–889. 17412921 10.1126/science.1138684

[39] Fukuda M, Kojima T, Kabayama H, Mikoshiba K (1996) Mutation of the pleckstrin homology domain of Bruton's tyrosine kinase in immunodeficiency impaired inositol 1,3,4,5-tetrakisphosphate binding capacity. J Biol Chem 271: 30303–30306. 8939985 10.1074/jbc.271.48.30303

[40] Miller AT, Berg LJ (2002) Defective Fas ligand expression and activation-induced cell death in the absence of IL-2-inducible T cell kinase. J Immunol 168: 2163–2172. 11859102 10.4049/jimmunol.168.5.2163

