# Supplementary figures and images for: Inhibition of the Inositol Kinase Itpkb Augments Calcium Signaling in Lymphocytes and Reveals a Novel Strategy to Treat Autoimmune Disease
Source: PLoS One. 2015 Jun 29;10(6):e0131071. doi: 10.1371/journal.pone.0131071 (PMC4488288; doi:10.1371/journal.pone.0131071)

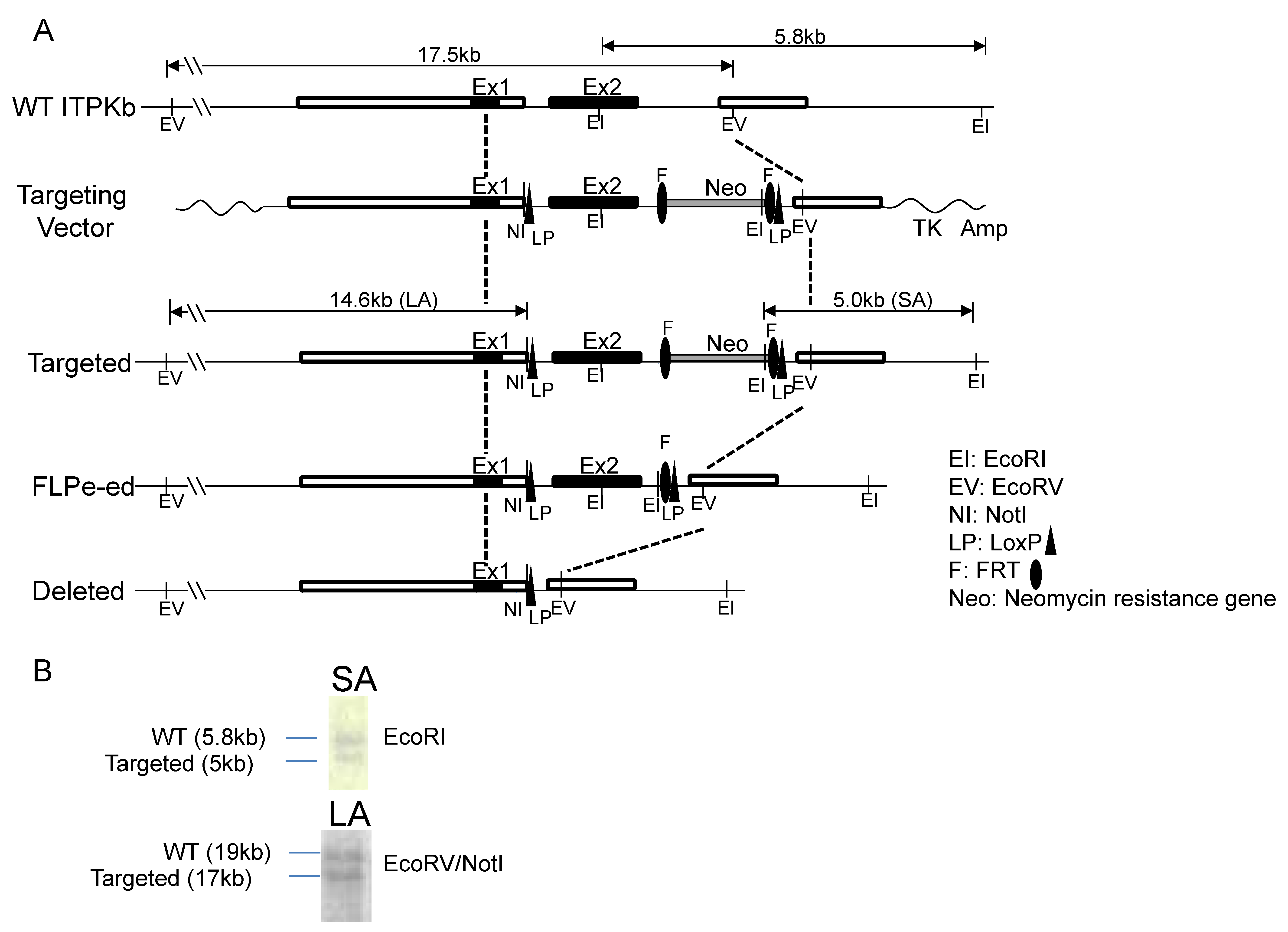

Supplement: S1 Fig — The gene targeting strategy for generation of the Itpkb conditional knockout where mice whose Itpkb exon 2 is flanked by two loxP sequences where crossed to mice transgenic for a tamoxifen-inducible Cre recombinase, to produce Itpkb fl/fl Cre + mice and Itpkb +/+ Cre + control mice (A). A targeting vector containing LoxP (LP, indicated by triangles) sites flanking Itpkb exon 2 was generated and the structure is shown with the indicated restriction enzyme sites (EcoRI, EI; EcoRV, EV; NotI, NI), Neo cassette, and FRT sites (indicated by ovals). The lengths of the predicted fragments following restriction enzyme digestion are also noted. The complete floxed allele was obtained after excision of the FRT-flanked neo cassette through crossing to FLPeR transgenic mice. The structure of the deleted allele obtained after Cre-mediated excision of the floxed region is also sketched. (B) DNA from the targeted CJ7 ES cells was digested with the indicated restriction enzymes and subjected to southern blot analysis with either a probe recognizing the short arm (SA) or long arm (LA). The short arm of the targeted allele yields a 5kb fragment upon EcoRI digestion. Similarly, the long arm of the targeted allele yields a 17kb fragment upon EcoRV and NotI digestion. (TIFF) [file pone.0131071.s001.tiff]

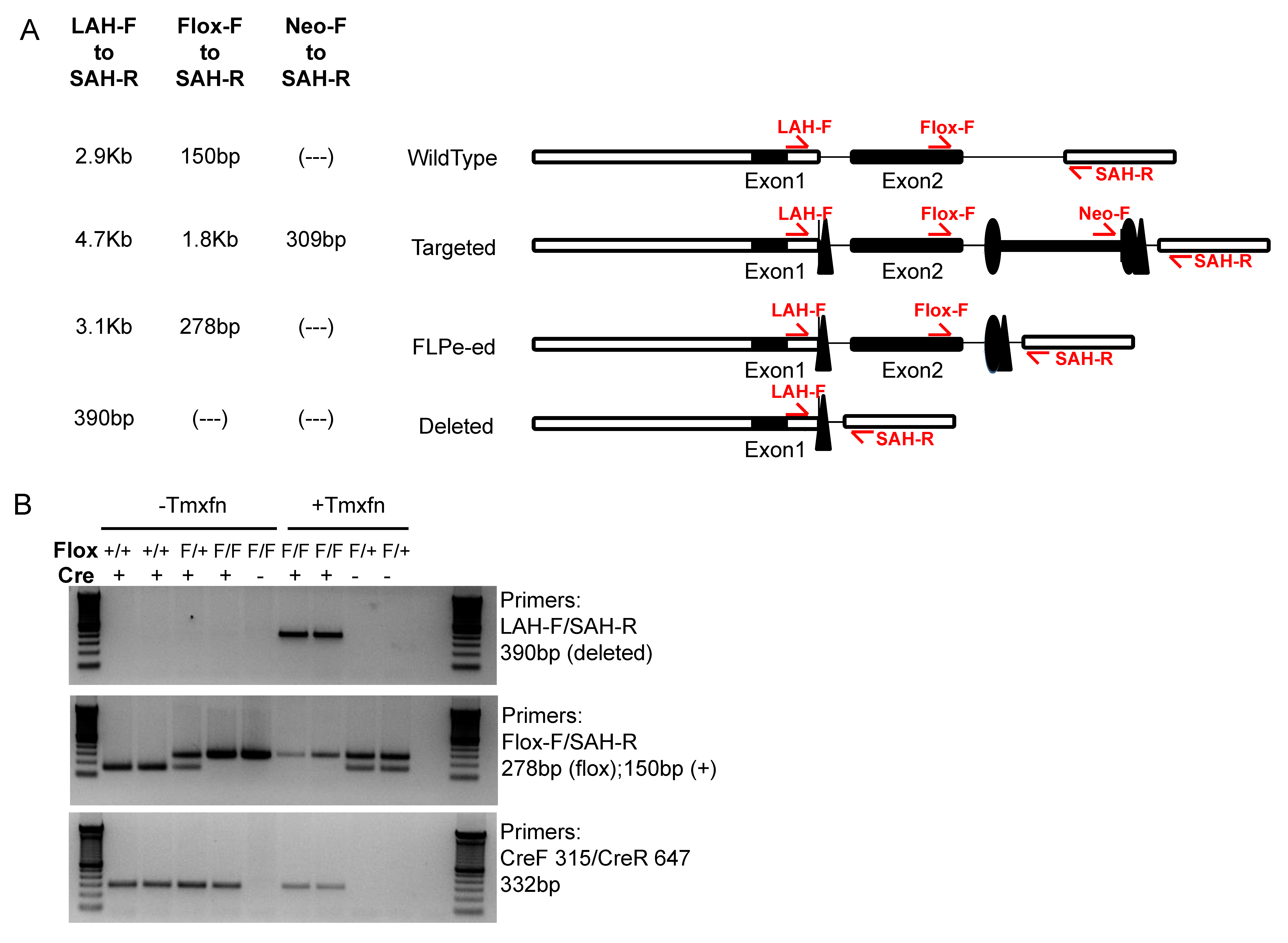

Supplement: S2 Fig — A schematic of lengths of the expected PCR products (A) are shown. Tail DNA from mice with the indicated genotypes that were either untreated or tamoxifen-treated, and representative PCR results are shown (B). (TIFF) [file pone.0131071.s002.tiff]

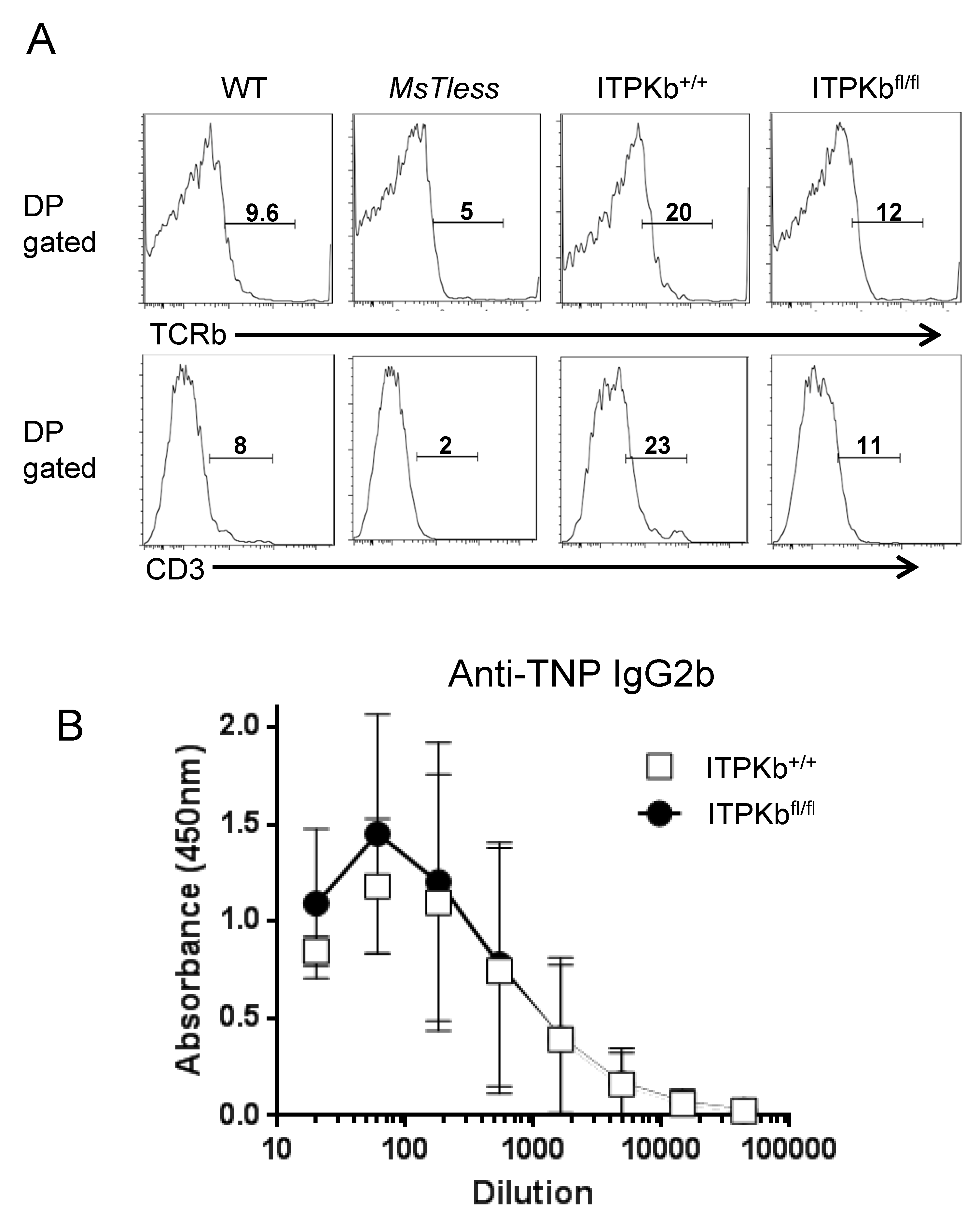

Supplement: S3 Fig — (A) Flow cytometry of thymocytes from WT, Itpkb -/-, Itpkb +/+ Cre +, and Itpkb fl/fl Cre + mice stained with antibodies to CD4, CD8, TCRb, and CD3. CD4+CD8+ cells were gated, and the percentage of cells expressing TCRb (top) or CD3 (bottom) is shown. The numbers in the plots indicate the percentages of each gated population. (B) Sera from WT and Itpkb fl/fl mice that were immunized with the T-independent antigen, TNP-Ficoll in Fig 2A, were tested for TNP-specific IgG2b antibody levels by ELISA on day 12 post-immunization. Data shown are one representative experiment (**, P < 0.01). (TIFF) [file pone.0131071.s003.tiff]

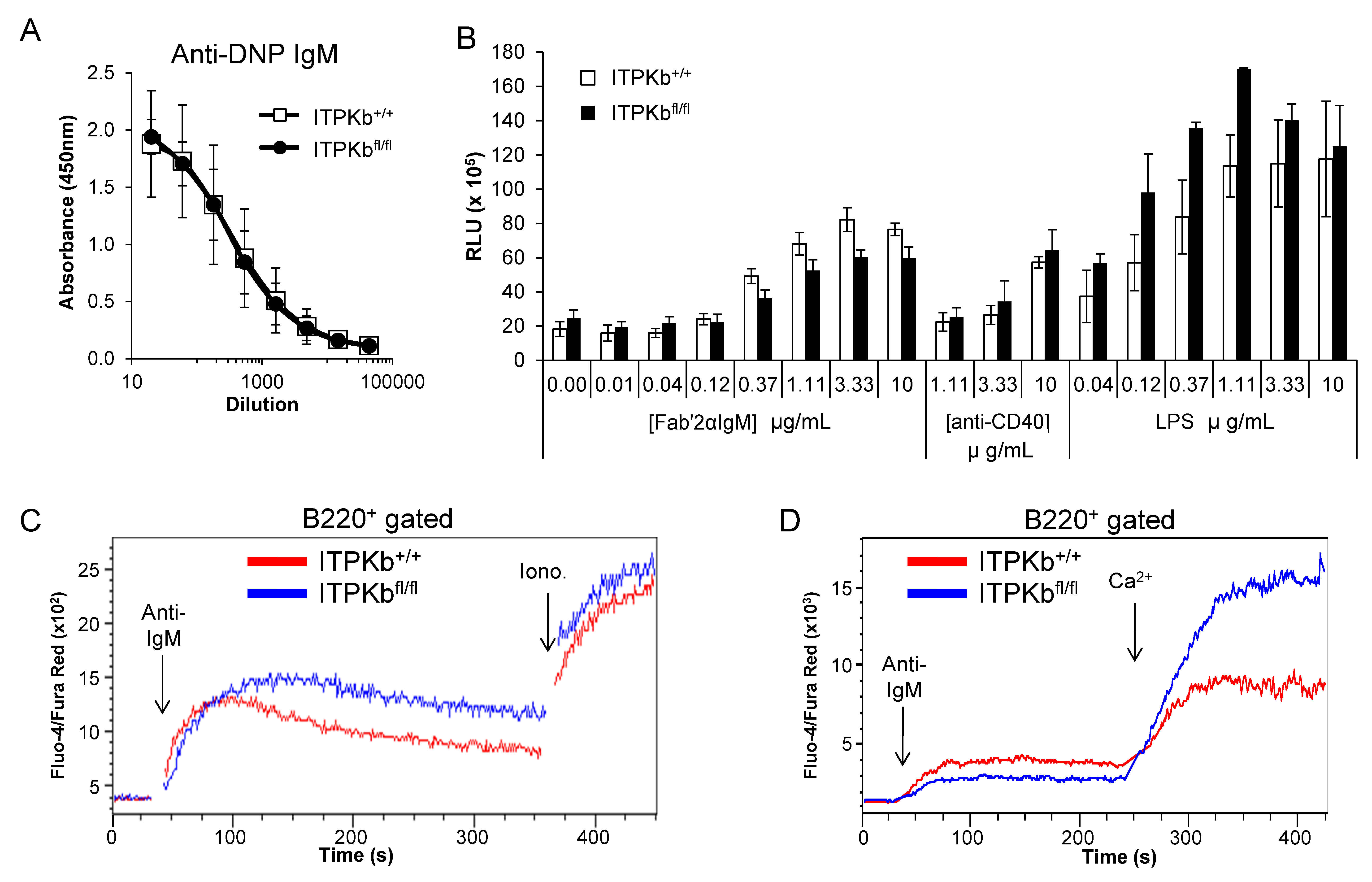

Supplement: S4 Fig — (A) B220+ cells were stimulated with various concentrations of F(ab’)2 anti-IgM, anti-CD40, or LPS, and proliferation was measured by Cell Titer Glo. (B) Splenocytes gated on B220 were stimulated with F(ab’)2 anti-IgM in the presence of exogenous calcium(C), or in the absence of exogenous calcium, followed by calcium re-addition (D). Data is shown as the mean fluorescent ratio of Fluo-3 and Fura-Red. The data are representative of five independent experiments. (TIFF) [file pone.0131071.s004.tiff]

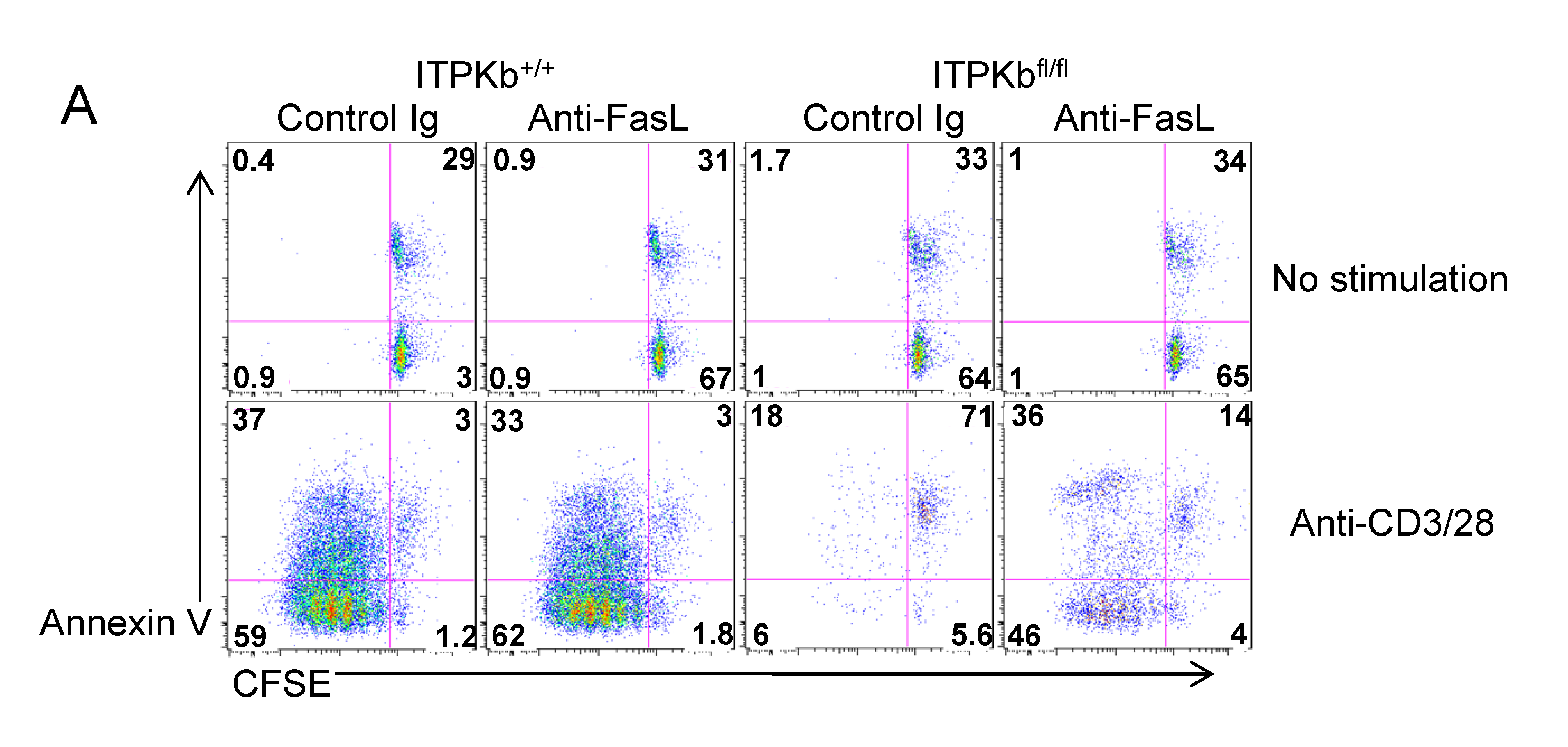

Supplement: S5 Fig — (A) Purified CD4+ cells were labeled with CFSE and stimulated with anti-CD3/28 beads in the presence of anti-FasL or an isotype control Ig. 72 hours following stimulation, CFSE dilution versus Annexin V staining was followed to determine whether Annexin V positivity required cell division. Numbers in the top right quadrant indicate the percentage of cells that died prior to cell division. Data shown are representative of four independent experiments. (TIFF) [file pone.0131071.s005.tiff]

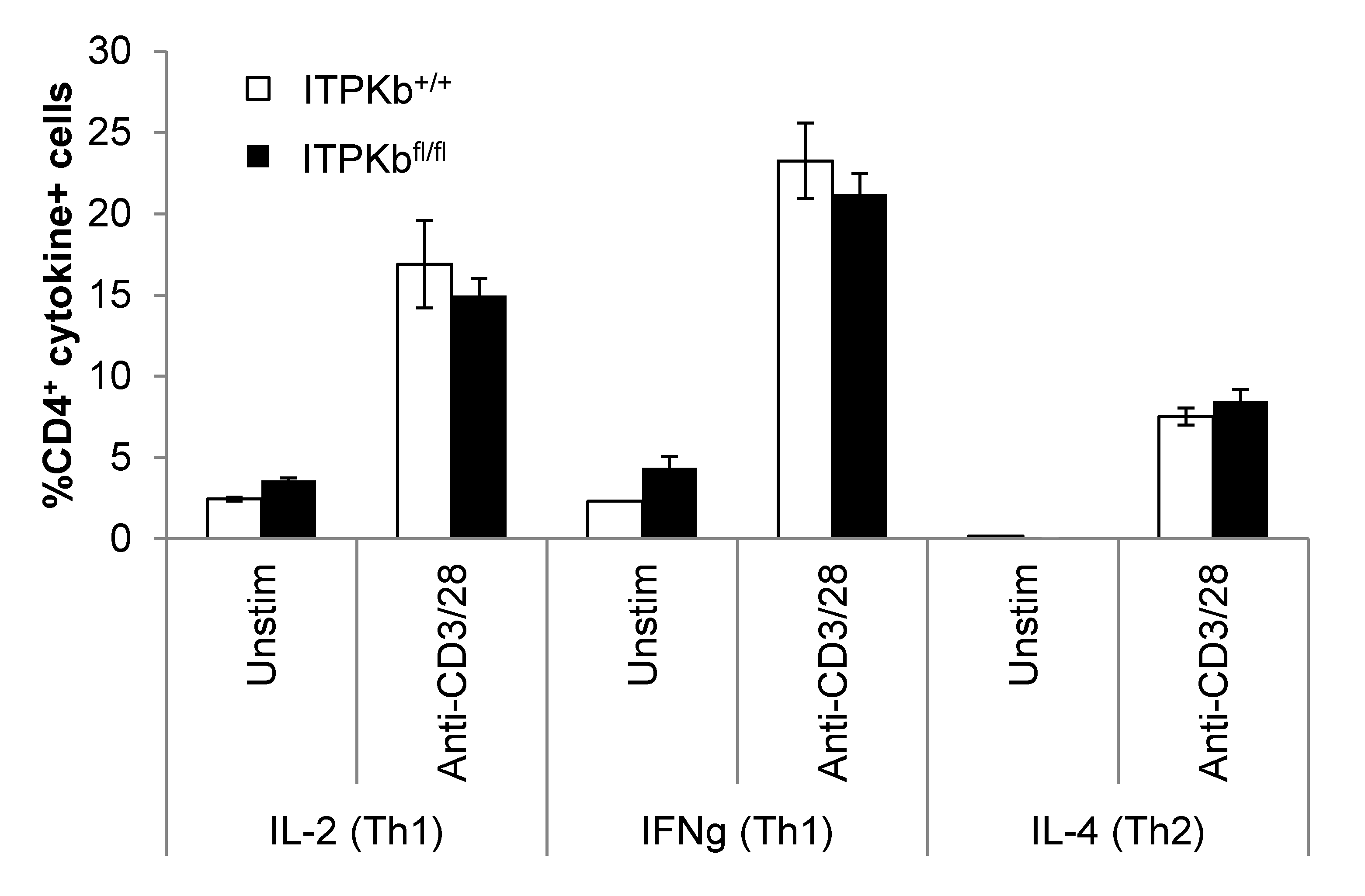

Supplement: S6 Fig — Itpkb-deficient T cells which survive primary stimulation do not possess any cytokine defects upon secondary stimulation. WT and Itpkb-deficient CD4+ T cells were stimulated with anti-CD3/28 beads in either Th1- or Th2-skewing conditions in the presence of exogenous IL-2. After 6 days in culture, live cells were re-stimulated and stained intracellularly for either IL-2 and IFNγ (Th1 cells) or IL-4 (Th2 cells). The bar graph represents the percentage of CD4+ cells which are positive for the respective cytokine. Data shown is representative of three independent experiments. (TIFF) [file pone.0131071.s006.tiff]

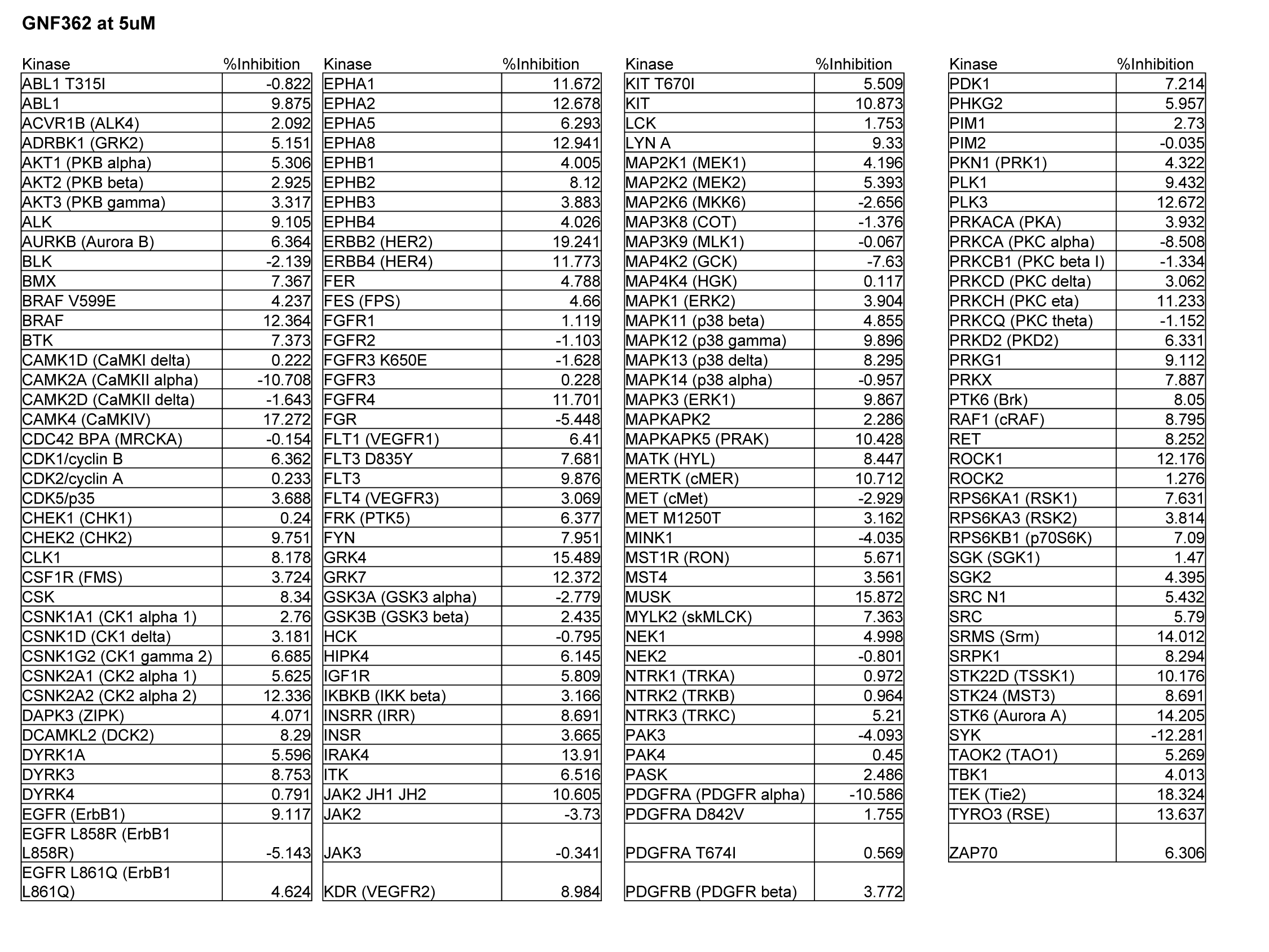

Supplement: S7 Fig — The percent of kinase inhibition at a concentration of 5μM is shown. (TIFF) [file pone.0131071.s007.tiff]

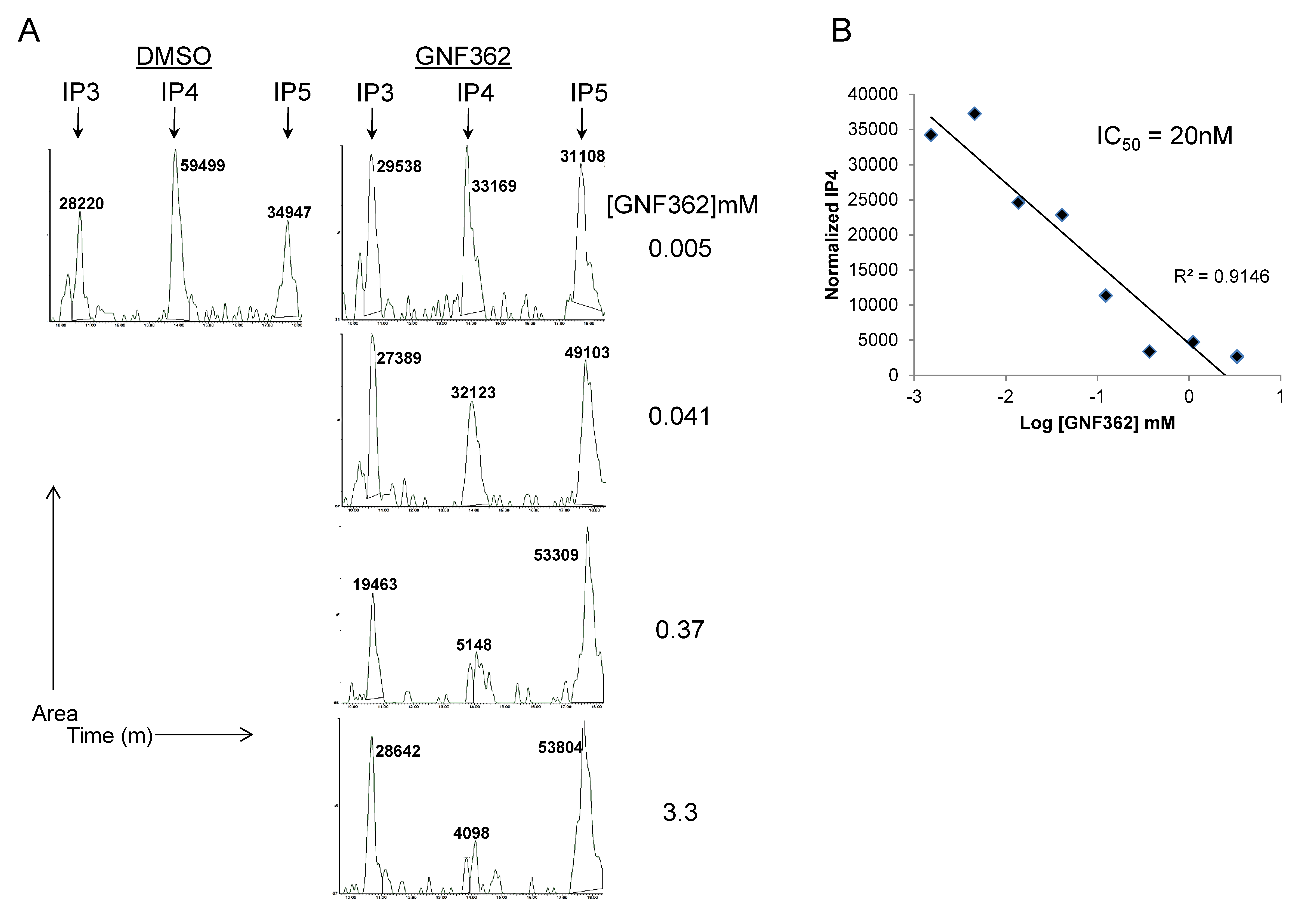

Supplement: S8 Fig — Jurkat T cells were labeled with 3H-myo-inositol and activated through the T cell receptor for 5 minutes. The inositol phosphates IP3, IP4, and IP5 were resolved by HPLC using an in-line β-ram detector. Raw HPLC traces from cells stimulated with anti-CD3 + anti-CD28 in the absence or presence of GNF362 are shown in (A). The area under the peaks corresponding to IP4 and IP5 were quantified, and data was normalized to IP5 levels, as this remained unchanged with stimulation. Normalized IP4 levels as a function of GNF362 concentration with an IC50 of 20nM is shown in (B). Data shown is one representative experiment. (TIFF) [file pone.0131071.s008.tiff]

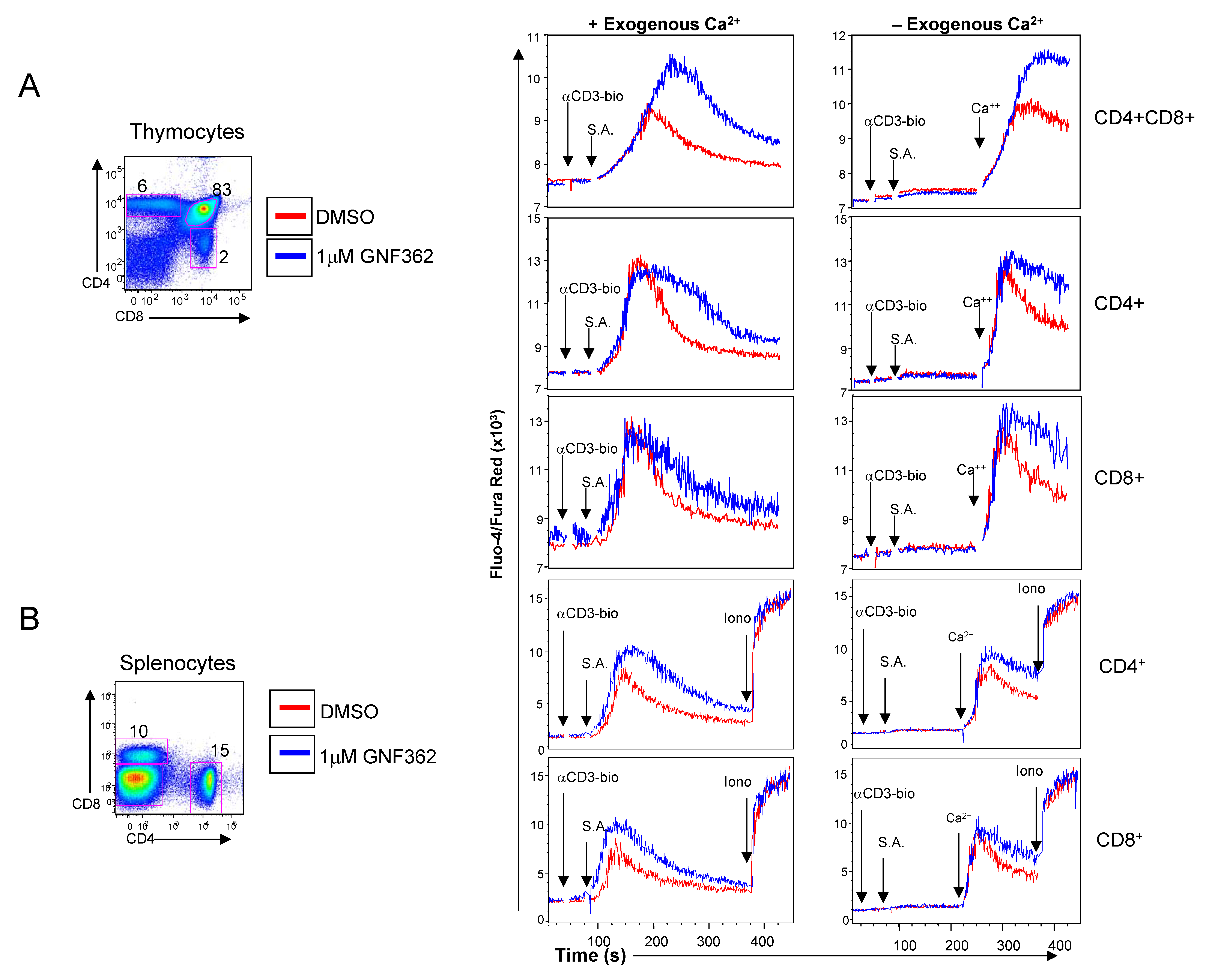

Supplement: S9 Fig — The effect of GNF362 on Ca2+ responses was measured using the Ca2+ sensitive dyes Fluo-4 and Fura Red following TCR-mediated cross-linking either in the presence or absence of exogenous Ca2+. (A) CD4+8+, CD4+, or CD8+ thymocytes pre-incubated with DMSO or 1μM of GNF362, were treated with anti-CD3-biotin, followed by cross-linking with streptavidin in the presence of exogenous calcium (left column), or in the absence of exogenous calcium, followed by calcium re-addition to examine SOC channel function (right column). (B) Similarly, CD4+ or CD8+ splenocytes were stimulated in the same fashion after pre-incubation with GNF362. Data is shown as the mean fluorescent ratio of Fluo-3 and Fura-Red. The data shown are representative of three independent experiments. (TIFF) [file pone.0131071.s009.tiff]

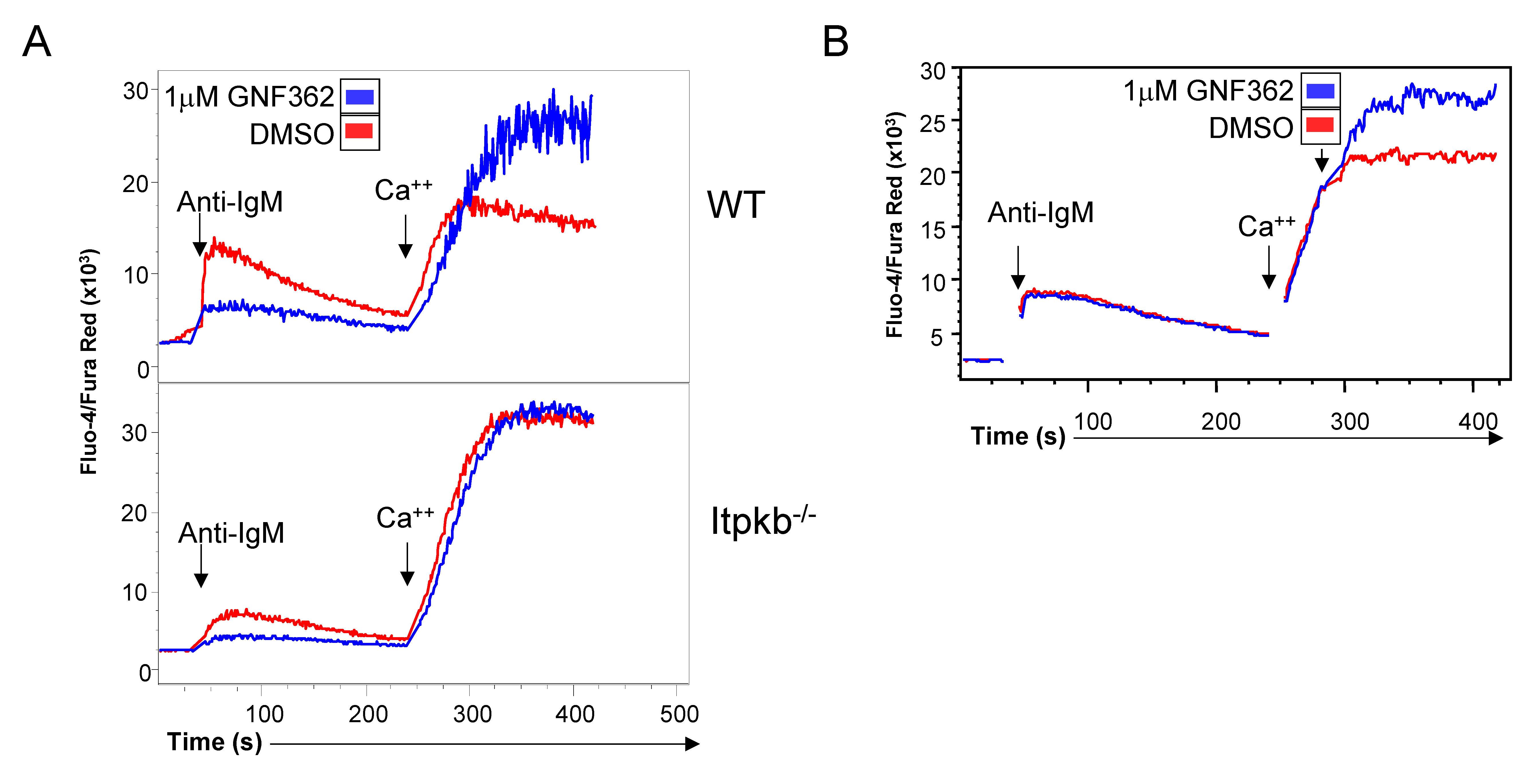

Supplement: S10 Fig — (A) To demonstrate Itpkb-dependency of GNF362, wild type (top panel) and Itpkb -/- (bottom panel) B cells pre-incubated with DMSO or 1μM of GNF362 were stimulated with anti-IgM in the absence of exogenous calcium, followed by calcium re-addition. (B) To examine the effect of GNF362 on SOC entry more directly, wild type B cells were stimulated with IgM in the absence of calcium, followed by calcium re-addition. DMSO or 1μM GNF362 were added to cells after calcium re-addition. Data is shown as the mean fluorescent ratio of Fluo-3 and Fura-Red. The data shown are representative of 3 independent experiments. (TIFF) [file pone.0131071.s010.tiff]

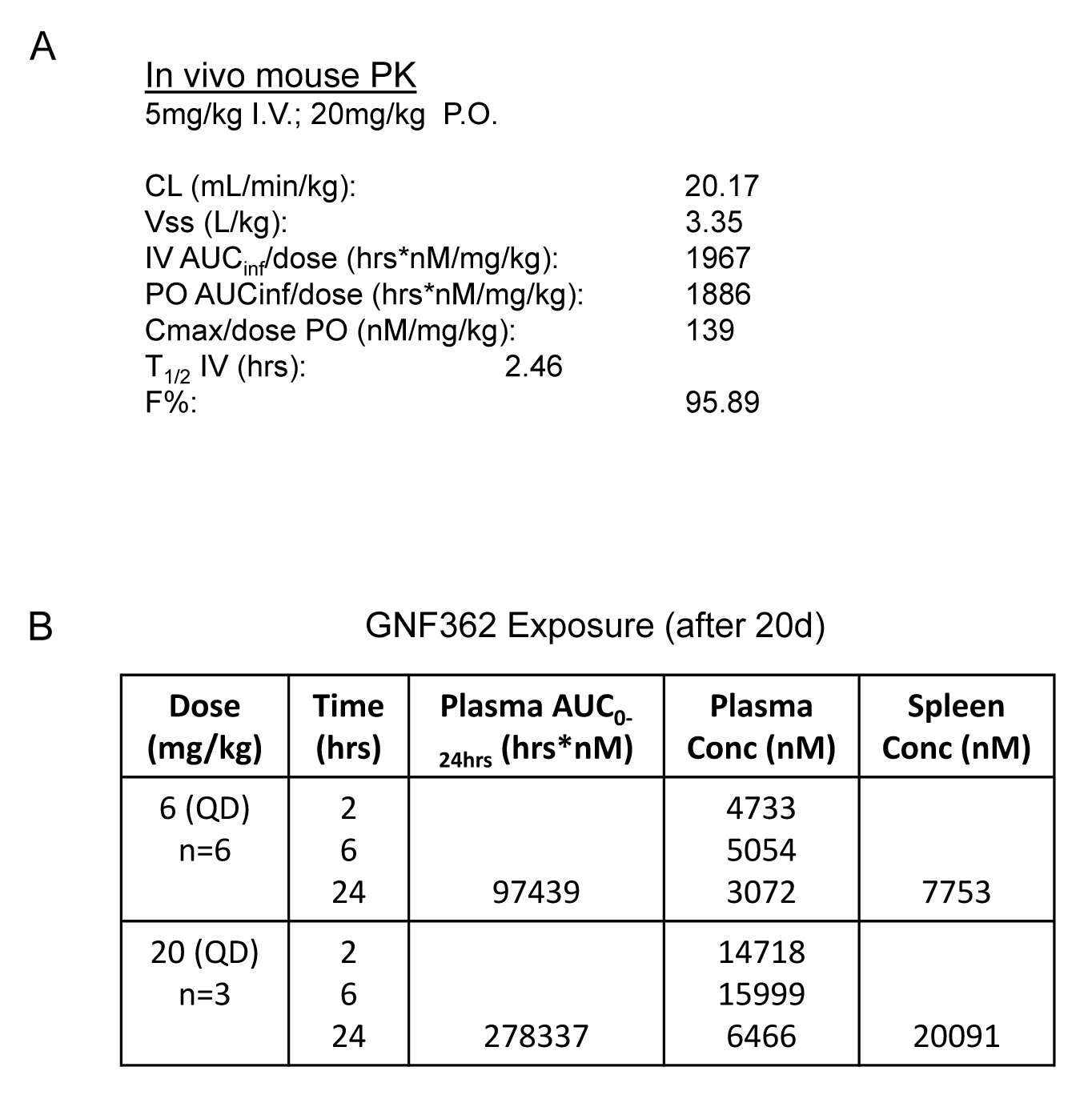

Supplement: S11 Fig — (A) Mice were dosed at 5mg/kg I.V., or 20mg/kg P.O., and bled at various time points. GNF362 levels in plasma were determined by mass spectrometry. Compound clearance (CL), volume distribution (Vss), total exposure (area under the curve, AUC), Cmax, and in vivo half-life (T1/2) were calculated. (B) Mice were dosed with GNF362 twice a day (QD) for 20 days at either 6 or 20 mg/kg. Compound levels in plasma were determined at 2, 6, and 24hrs following the last dose. In addition, at the termination of the study, compound levels in the spleen were determined at 24hrs following the last dose. Data is representative of at least five independent experiments. (TIFF) [file pone.0131071.s011.tiff]

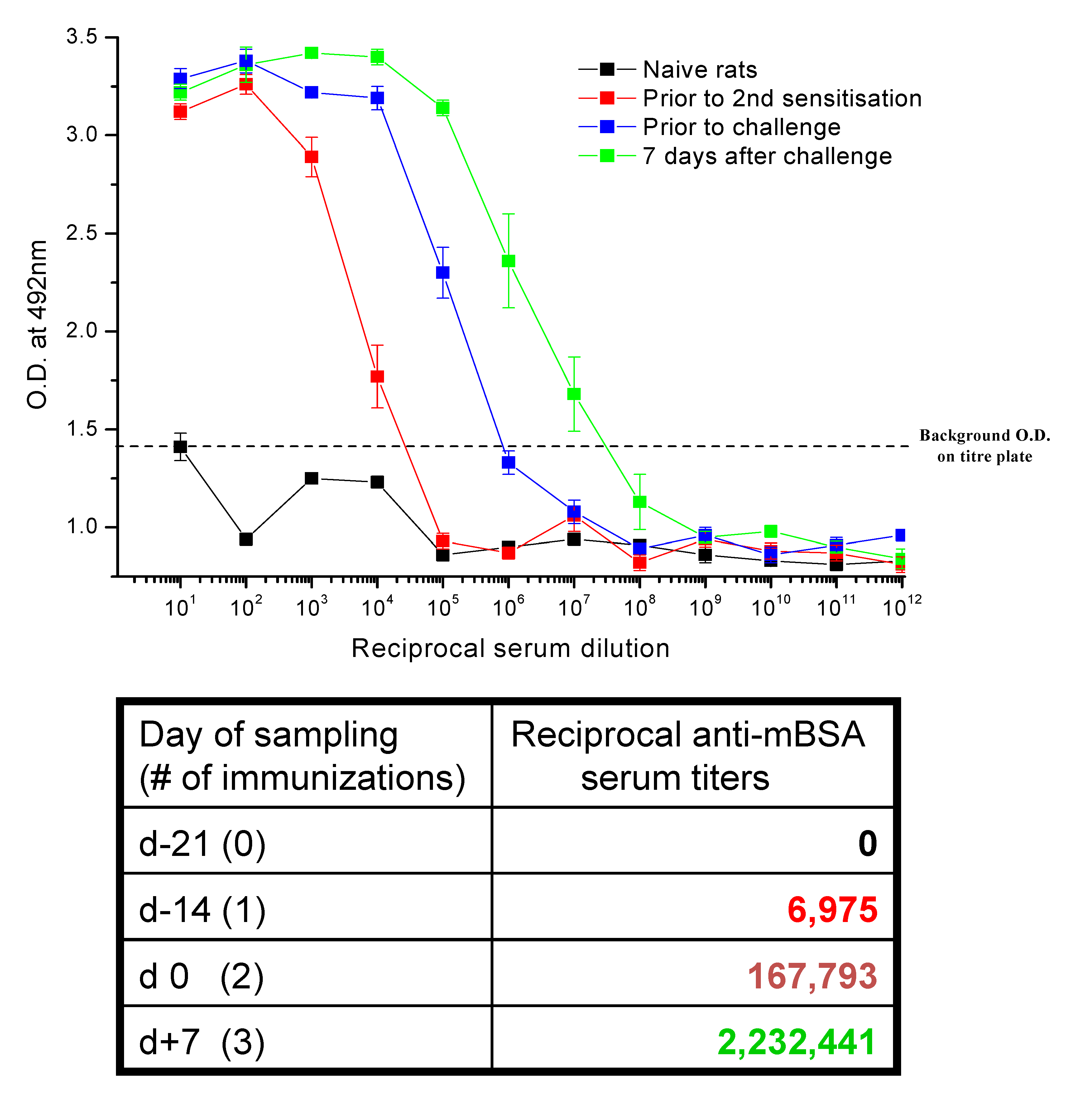

Supplement: S12 Fig — Repeated immunization with mBSA increases antibody levels. Rats were immunized intra-dermally with mBSA on Days -21 and -14, followed by an intra-articular challenge on Day 0. Antibody titers to mBSA at Days -21, -14, 0, and +7 were determined by ELISA. Data shown is one representative experiment. (TIFF) [file pone.0131071.s012.tiff]

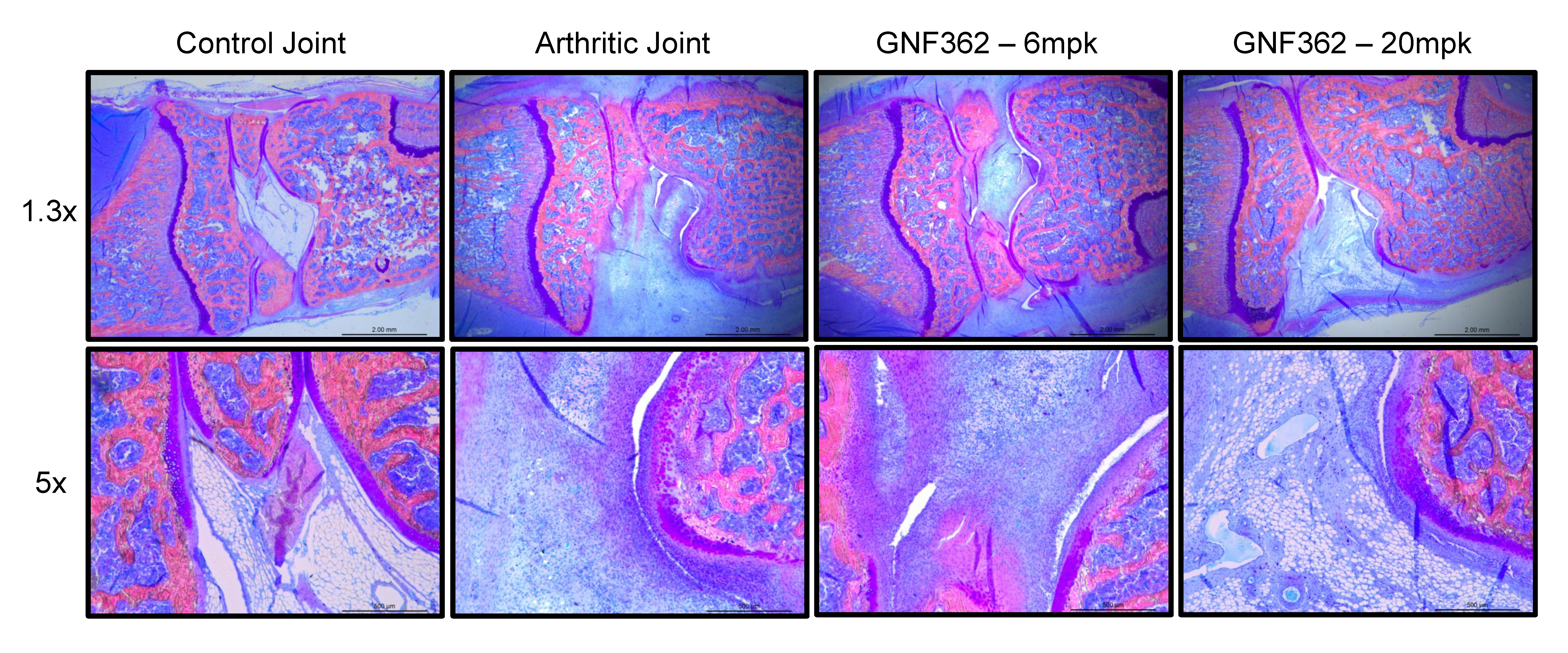

Supplement: S13 Fig — Following the termination of the arthritis study, knee joints from each group were removed and paraffin sections were subjected to histological analysis following Giemsa and Saffranin O staining. Representative histopathology data are shown at 1.3x and 5x magnification. Data shown is one representative experiment. (TIFF) [file pone.0131071.s013.tiff]
